# Feedback regulation of Notch signaling and myogenesis connected by MyoD–Dll1 axis

**DOI:** 10.1371/journal.pgen.1009729

**Published:** 2021-08-09

**Authors:** Haifeng Zhang, Renjie Shang, Pengpeng Bi

**Affiliations:** 1 Center for Molecular Medicine, University of Georgia, Athens, Georgia, United States of America; 2 Department of Genetics, University of Georgia, Athens, Georgia, United States of America; Indiana University Purdue University at Indianapolis, UNITED STATES

## Abstract

Muscle precursor cells known as myoblasts are essential for muscle development and regeneration. Notch signaling is an ancient intercellular communication mechanism that plays prominent roles in controlling the myogenic program of myoblasts. Currently whether and how the myogenic cues feedback to refine Notch activities in these cells are largely unknown. Here, by mouse and human gene gain/loss-of-function studies, we report that MyoD directly turns on the expression of Notch-ligand gene Dll1 which activates Notch pathway to prevent precautious differentiation in neighboring myoblasts, while autonomously inhibits Notch to facilitate a myogenic program in Dll1 expressing cells. Mechanistically, we studied *cis*-regulatory DNA motifs underlying the MyoD–Dll1–Notch axis *in vivo* by characterizing myogenesis of a novel E-box deficient mouse model, as well as in human cells through CRISPR-mediated interference. These results uncovered the crucial transcriptional mechanism that mediates the reciprocal controls of Notch and myogenesis.

## Author summary

The formation of skeletal muscle tissue during development and regeneration is orchestrated by controlling the expression levels of muscle-specific transcriptional factors including MyoD gene. Previous studies have identified the key function of Notch signaling, an evolutionarily conserved cell-cell communication pathway, in blocking the expression of MyoD and the generation of functional muscle cells. Therefore, at the beginning of myogenesis, the activity of Notch pathway needs to be downregulated in order to promote MyoD expression thus the induction for expression of muscle structural genes. Here, we identified a key regulatory mechanism by which MyoD directly induces the transcription of Dll1 gene which encodes a classical Notch ligand. Interestingly, Dll1 can dampen Notch cell-autonomously to promote muscle cell differentiation while activate Notch in neighboring cells to block their myogenic differentiation. The discovery of the MyoD–Dll1–Notch gene control axis fills the knowledge gap for our understanding of the molecular regulation of myogenesis.

## Introduction

Skeletal muscle accounts for around 40% of adult human body weight. Myogenesis involves a series of events that begins with the specification of muscle lineage by master transcriptional regulators, including Pax7 and Muscular Regulatory Factor (MRF), followed by the expression of a vast number of genes that establish muscle structure and function [1–6]. In adult tissue, muscle stem cells normally remain in a quiescent state, but can be promptly activated by injury, following which the stem cells enter cell-cycle to generate a pool of precursors which either differentiate to repair myofiber or self-renew to replenish stem cell pool [7, 8]. The precise control of these diverse states of muscle stem cell is key for proper tissue homeostasis [9–11].

Notch signaling is an ancient intercellular communication mechanism that determines the cell-fate for various tissue types in metazoan [12–15]. Alterations of this pathway underline a spectrum of disease and cancer [16–20]. Transduction of Notch signaling is initiated upon binding of a Notch receptor to a ligand located on a neighboring cell [14, 21]. Notch ligands are members of the DSL (Delta/Serrate/LAG-2) family protein that include Delta-like (Dll1, Dll3, Dll4) and Jagged (Jag1, Jag2) in mammals. Endocytosis of Notch-bound ligand generates a mechanical pulling force that drives conformational change of the Notch receptor [22]. This facilitates subsequent proteolytic cleavage of Notch receptor and produces Notch intracellular domain (NICD) [23]. As a transcriptional activator, NICD then translocates to nucleus where it binds with Rbpj and recruits a transcriptional complex to activate the expression of downstream target including Hairy/enhancer of split (Hes) and Hes-related with YRPW motif (Hey) family genes [24]. Despite the simplicity in design, the biological function of Notch signaling is highly context-dependent [12, 25]. This is in part due to versatile ligand-utilizations for signaling in various biological processes [25, 26].

In skeletal muscle, Notch is popularly known as a potent inhibitor of myogenic differentiation [27–31]. Genetic studies also unveiled a key paradigm of this signaling pathway in enforcing the quiescent state of muscle stem cells [30, 32–35]. Of note, deletion of Rbpj or Notch-ligand Dll1 in mouse resulted in depletion of muscle progenitor cells accompanied by severe muscle hypotrophy and failure of muscle regeneration [30, 36–38]. On the other hand, constitutive activation of Notch in myocytes is sufficient to induce cellular dedifferentiation that re-expression of stem-cell markers [39]. Notch signaling is also essential to build a specialized microenvironment or niche that controls muscle stem cell function [40–42]. In the proximity to stem cell, endothelial cells from microvasculature can supply Notch-ligand Dll4 that activates Notch and maintains a quiescent state of muscle stem cells [43]. Similarly, differentiating muscle cell can also convey a self-renewing signal by providing Dll1 [44, 45]. Although Dll1 and Dll4 appeared to exert similar function for skeletal muscle cell in mouse, opposing function of these ligands in regulation of myogenesis were identified in chick somites [46].

Currently, it remains largely unknown whether and how the core myogenic program feedback to restrain Notch activity; and how does this reciprocal regulation determine the differentiation dynamics of myoblast. Here, by gene gain/loss-of-function studies in human and mouse cells, we unraveled the crucial role of MyoD in controlling Dll1 expression and Notch activity. Strikingly, deletion of MyoD in mouse or human myoblast abolished Dll1 expression, whereas forced expression of MyoD robustly induced Dll1 transcription in fibroblast. Utilizing a novel line of mutant mouse, we also probed the *cis*-regulatory element whereby MyoD controls Dll1 expression. Employing heterologous cell-mixing experiment, we report the *cis*-inhibitory and *trans*-activation roles of Dll1 on Notch transduction whereby divergent myogenic programs can be established among the subpopulations of myoblast. These results provide an insight to the evolutionarily conserved control mechanism of myogenesis for mouse and human.

## Results

### Strong induction of Notch ligand Dll1 expression during human and mouse myogenesis

**Fig 1:**
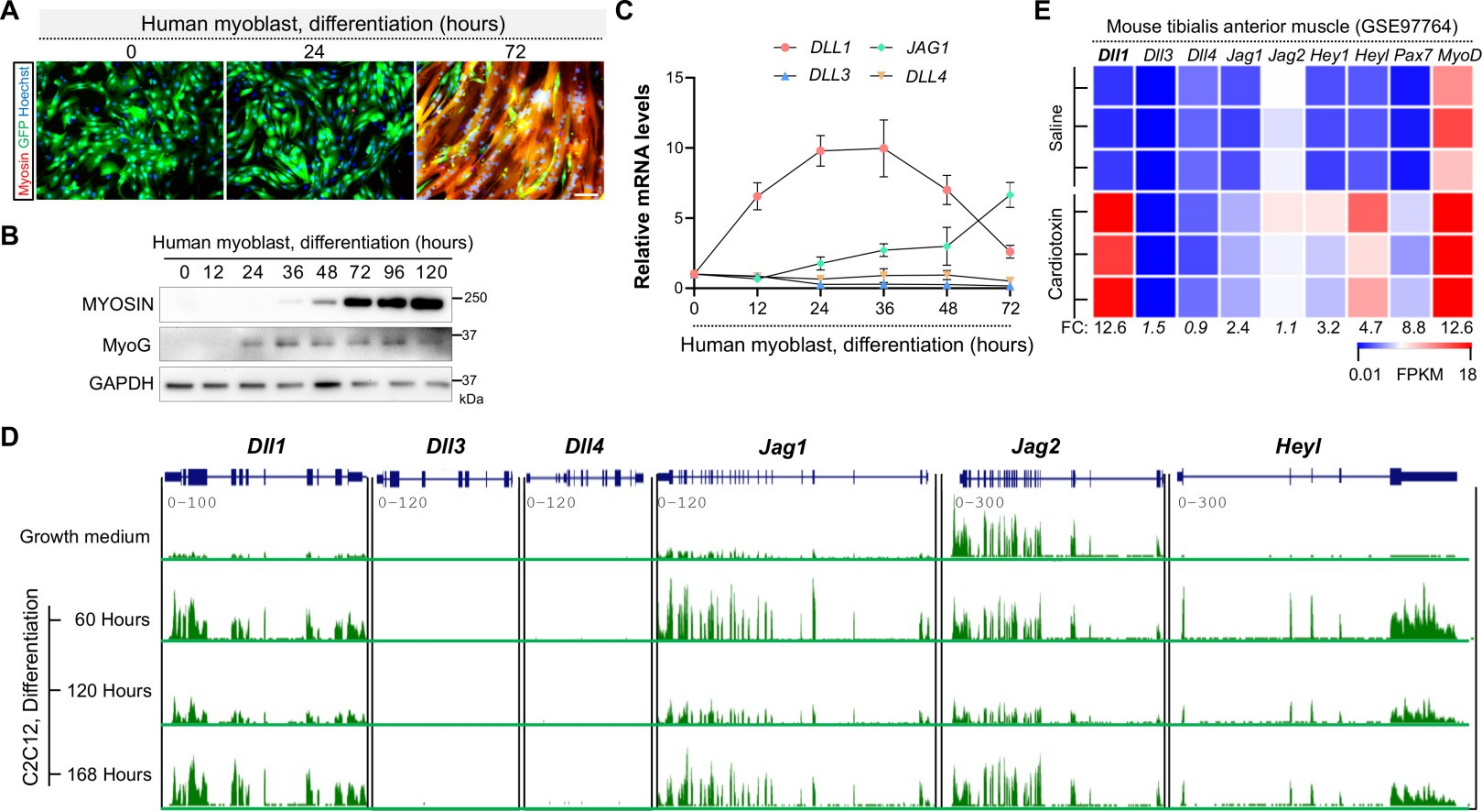
Robust inductions of *Dll1* expression by myogenic cues in both mouse and human cells. (**A**) Human myoblasts display high myogenic and fusogenic potentials in culture. Cells were labelled by GFP to visualize the syncytium at early stages of differentiation. Scale bar, 100 μm. (**B**) Western blotting results of myosin (MF20 antibody) and Myogenin (MyoG) in human myoblasts at various stages of differentiation. (**C**) qPCR results of Notch ligand genes during human myoblast differentiation. The Ct values for each gene at growth-culture condition (time 0) are *Dll1* (25.6), *Dll3* (26.8), *Dll4* (32.4) and *Jag1* (28.6). *Jag2* expression was not detected. (**D**, **E**) RNA sequencing results of Notch ligand genes and Notch target *Heyl* during mouse myoblast differentiation (D, GSE20846) and muscle regeneration (E, GSE97764). CTX (cardiotoxin) samples were from day 3 post injury. FC, expression fold change.

Notch ligands play either redundant or divergent functions depending on the biological context [26, 47]. To understand which Notch ligand(s) may participate the human myogenic program, we performed gene expression analyses in low-passage human myoblasts that were derived from a healthy donor as described previously [48, 49]. Of note, these cells display robust myogenic and fusogenic potentials (Fig 1A). Shortly after three days’ induction, huge myosin+ syncytia that contained hundreds nuclei can be observed (Fig 1A), accompanied by prompt inductions of myogenin (MyoG) and myosin expression (Fig 1B). Therefore, these human myoblasts can serve as an ideal model to dissect the genetic mechanism of human myogenesis.

Among all five Notch ligands, expression of *DLL1* was the highest induced and peaked at 36 hours post human myoblast differentiation (Fig 1C). Its expression was downregulated thereafter (Fig 1C). By comparison, expression of *JAG1* was induced toward later stages of myoblast differentiation. In parallel, we also examined the expression patterns of Notch ligand genes during mouse myogenic differentiation *in vitro* and muscle regeneration *in vivo* by querying RNA-seq datasets generated from previous studies [50, 51]. Similar with human data, expression of *Dll1* and *Jag1* were promptly induced upon differentiation of mouse myoblasts (Fig 1D), though only *Dll1* showed a dramatic increase during muscle regeneration (Fig 1E). Expression of canonical Notch target gene *Heyl* was also significantly upregulated in these myogenic conditions (Fig 1D and 1E). These results indicate that Dll1 may play an evolutionarily conserved role in controlling myogenesis for the various mammal species.

### DLL1 transactivates Notch signaling which inhibits human myoblast differentiation

**Fig 2:**
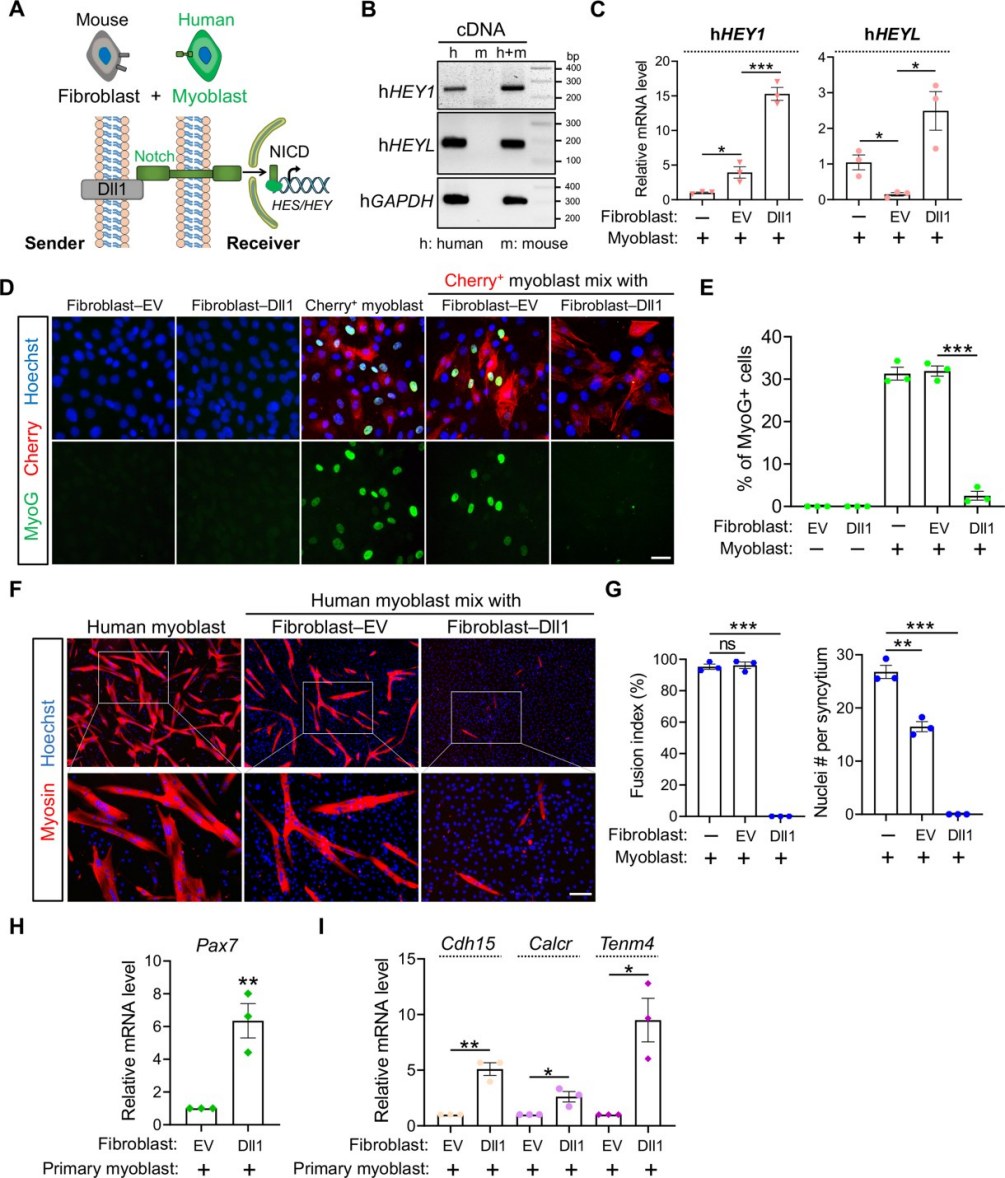
Dll1 transactivates Notch which inhibits human myogenic differentiation. (**A**) Schematics of heterologous cell-mixing assay and Notch signaling pathway. (**B**) DNA electrophoresis results that validated the specificity of human qPCR primers. (**C**) qPCR results of human *HEY1* and *HEYL* using primers tested in B. Cells were differentiated for 24 hours after mixing. EV: empty control vector. The presence or absence of indicated cell types is indicated by plus or minus sign, respectively. *n* = 3. (**D**) Fluorescence images of mouse fibroblasts and human myoblasts in separate or mixing-culture conditions. Human myoblasts were labelled by red fluorescence protein Cherry; mouse fibroblasts (10T1/2) were infected by control (EV) or Dll1 expressing retroviruses. Cells were differentiated for 24 hours. Scale bar, 25 μm. (**E**) Quantification results of MyoG+ myoblasts as shown in D. *n* = 3. (**F**) Myosin immunostaining of human myoblasts that were differentiated for 96 hr. Scale bar, 100 μm. (**G**) Measurement of myoblast fusion as in F. *n* = 3. (**H**, **I**) qPCR results for genes in primary mouse myoblasts after mixing culture with fibroblasts. Data are means ± SEM. *P < 0.05, **P < 0.01, ***P < 0.001. ns, not significant.

Depending on the model of selection, activation of Notch by Dll1 was reported to either promote myogenesis in chicken [46] or inhibit myogenesis in mice [37, 44]. To probe the role of Dll1 in regulations of Notch signaling and human myogenesis, we set up a unique cell-mixing and gene expression assay (Fig 2A). First, we overexpress Dll1 in mouse fibroblasts which were later co-cultured with human myoblasts. RNA from the mixing culture was collected and used to measure Notch-target gene expression with human sequence-specific primers. This allows us to gauge Notch activity in human myoblasts without force separating them from fibroblasts. Given Notch activation strictly requires physical contact between adjacent cells, this experiment design avoids potential disruption of Notch signaling from scenarios where cells have to be first separated before gene expression analysis.

We selected Notch target genes HEY1 and HEYL to gauge Notch activity because they are abundantly expressed in human myoblasts while the species-specificity of qPCR primers were confirmed (Fig 2B). Through these experiments, we detected dramatic inductions of *HEY1* and *HEYL* expression in human myoblasts when co-cultured with mouse fibroblasts expressing Dll1 (Fig 2C), suggesting that Dll1 is capable of transactivating Notch pathway in human muscle cells.

We continued to dissect the biological function of Dll1 in human myoblasts by examining its impact on the myogenic differentiation. Human myoblasts were pre-labelled with red fluorescence protein Cherry and induced to differentiate after mixing with mouse Dll1+ fibroblasts. Notably, this co-culture scheme strongly inhibited myogenic differentiation evidenced by drastic reductions of MyoG and myosin expressions (Fig 2D–2F). Accordingly, myoblast fusion, a hallmark of skeletal myogenesis, was completely abolished when the cells were co-cultured with Dll1+ fibroblasts (Fig 2F and 2G).

Beyond the inhibition of myogenic differentiation, Notch signaling is also essential for maintaining the quiescence of muscle stem cells and the expression of Pax7, a faithful marker and master regulator of muscle stem cells [52]. Because the immortalized human myoblasts do not express Pax7, we chose primary mouse myoblasts to examine a role of Dll1-transduced Notch signaling on the expression of muscle stem cell markers. As expected, when these cells were mixed with Dll1+ fibroblasts, Pax7 expression was significantly increased, along with other markers that are either exclusively (*Calcr*, *Tenm4*) or abundantly (*Cdh15*) expressed by quiescent muscle stem cells (Fig 2H and 2I).

### Dll1 *cis*-inhibits Notch and promotes human myogenic differentiation

Dll1 expression can be promptly induced in myocytes by myogenic stimulation. Given the potent anti-myogenic activity of Notch, permissive Notch activations by signaling within Dll1+ myocytes would cause the termination myogenic differentiation. Therefore, we hypothesized that Dll1+ myocytes may employ an intrinsic mechanism to escape Notch activation in order to complete the myogenic program.

**Fig 3:**
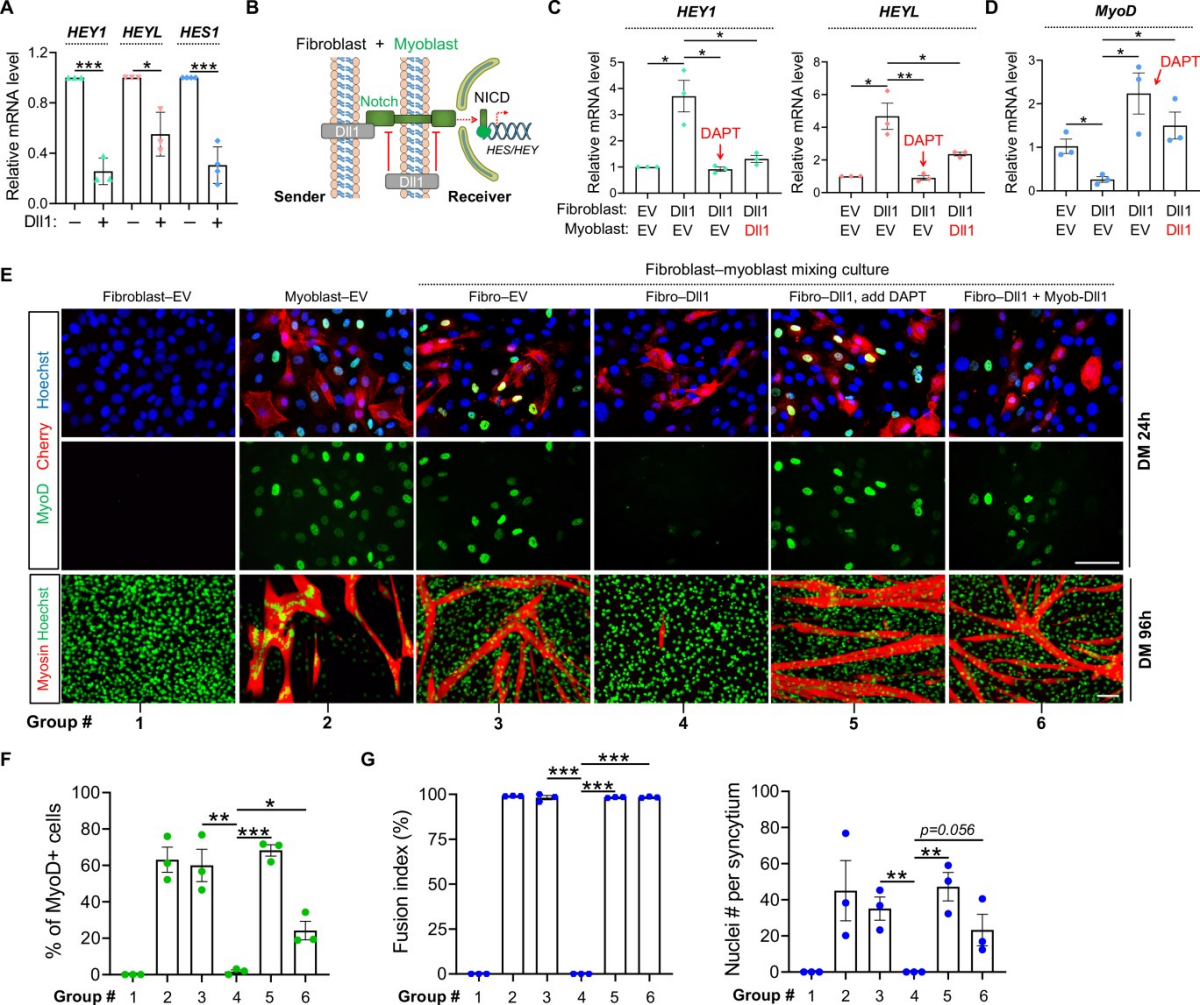
Dll1 *cis*-inhibits Notch signaling and promotes human myoblast differentiation. (**A**) qPCR results of Notch target genes in human myoblasts in response to Dll1 overexpression. Cells are cultured in growth medium. (**B**) Schematic of Notch signaling in heterologous cell-mixing assays for panels C–G. (**C**, **D**) Relative mRNA levels of Notch target genes *HEY1*/*HEYL* (C) and *MyoD* (D) in human myoblasts. Cells were differentiated for 24 hours. *n* = 3. (**E**) Fluorescence images of mouse fibroblasts (10T1/2) and human myoblasts in separate or mixing-culture conditions. Human myoblasts were labelled by red fluorescence protein Cherry; mouse fibroblasts or human myoblasts were infected by control (EV) or Dll1 expressing retroviruses. Cells were mixed at day-2 post infection followed by myogenic differentiation for 24 hours (top) or 96 hours (bottom). Scale bar, 100 μm. (**F**) Quantification results of MyoD+ cells as in E (top). *n* = 3. (**G**) Measurements of fusion index and nucleus number per syncytium of human myotubes as in E (bottom). Cells were differentiated for 96 hours. Treatments in groups 1–6 are same for panels E–G. *n* = 3. Data are means ± SEM. *P < 0.05, **P < 0.01, ***P < 0.001.

Intriguingly, forced expression of Dll1 in human myoblasts significantly reduced the expression of Notch target genes *HEY1*, *HEYL* and *HES1* by 75%, 45% and 70% respectively (Fig 3A). This result hints at a *cis*-inhibitory action of Dll1, i.e. ectopic ligand expression in signal-receiver cells blocked the signaling from senders, a paradigm that was previously reported in other developmental processes [53]. To prove this, we again employed heterologous cell-mixing experiment that can distinguish the *cis-* and *trans*-effects of Dll1 (Fig 3B). Consistently, overexpression of Dll1 in mouse fibroblasts can faithfully activate Notch in human myoblasts shown by inductions of *HEY1* and *HEYL* expression (Fig 3C), accompanied by drastic reductions of MyoD expression (Fig 3D–3F) and myoblast fusion (Fig 3E and 3G). As expected, such *trans-*acting effects of Dll1 were abolished by γ-secretase inhibitor DAPT (Fig 3E–3G, group 5 vs 4). Interestingly, similar to DAPT, forced expression of Dll1 in human myoblasts (signal receivers) also blocked Notch activation and restored MyoD expression, myogenic differentiation and myoblast fusion (Fig 3C–3G, group 6 vs 4). Together, these results reveal the *cis*-inhibitory role of Dll1 that blocks Notch activation while promotes myogenic differentiation.

### Deletion of *DLL1* from human myoblasts abolishes its *trans*-activating and *cis*-inhibitory effects on Notch signaling

**Fig 4:**
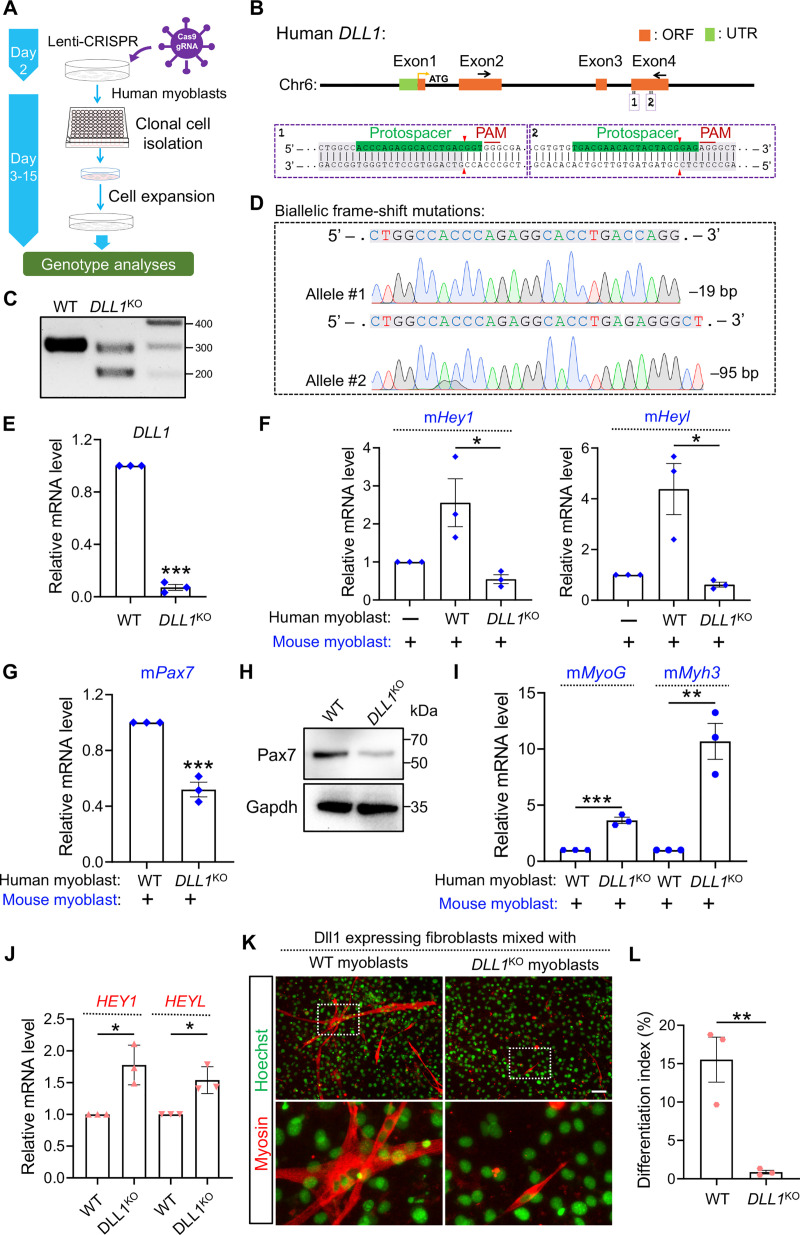
DLL1 loss-of-function study in human myoblasts. (**A**) Schematic of CRISPR/Cas9 approach to generate gene-knockout clones from human myoblasts. (**B**) Schematic of human *DLL1* gene structure and the sequences of gRNAs in exon 4. (**C**) DNA electrophoresis results of *DLL1* genotyping PCR. (**D**) Sanger sequencing analysis of human DLL1 genotyping PCR products. The premature stop-codon was detected in exon 5 and exon 7 for the –95 bp and –19 bp allele, respectively. (**E**) qPCR results of *DLL1* measured using primers located in exon 3 (forward) and exon 4 (reverse). *DLL1* has total 11 exons. (**F**, **G**) qPCR results for genes in mouse myoblasts that were co-cultured with human myoblasts of indicated genotypes. (**H**) Western blotting analysis of protein lysates from mixing culture of mouse/human myoblasts. Note that the immortalized human myoblasts do not express Pax7. (**I**) qPCR results for genes in mouse myoblasts that were co-cultured with human myoblasts of indicated genotypes. (**J**) qPCR results that measured HEY1 and HEYL expression in WT or DLL1^KO^ human myoblasts after co-culturing with Dll1+ fibroblasts. (**K**) Myosin immunostaining of human myoblasts. (**L**) Quantification of myosin+ cells as in panel K. *n* = 3. Data are means ± SEM. *P < 0.05, **P < 0.01, ***P < 0.001.

To examine the role of endogenous DLL1 in human myoblasts, we performed *DLL1* loss-of-function study by CRISPR/Cas9 mediated mutagenesis (Fig 4A). First, human myoblasts were transduced with lentiviruses that deliver the expression of Cas9 and one pair of guide RNA (gRNA) targeting the coding exon 4 of *DLL1* gene (Fig 4B). Later, we obtained myoblast clones that were expanded from single-cell isolations after CRISPR treatment (Fig 4A). Genotyping analysis by PCR (Fig 4C) and sequencing (Fig 4D) revealed biallelic frameshift mutations in one *DLL1* knockout clone (*DLL1*^KO^). Possibly due to nonsense-mediated decay of resultant messenger RNA, expression of *DLL1* in *DLL1*^KO^ myoblasts was reduced by 87%, compared to control clones transduced with only Cas9 (Fig 4E). As expected, the signaling of Notch from human to mouse cells in the mixing culture was compromised when DLL1 was deleted in human myoblasts (Fig 4F). The dampened Notch activity in mouse myoblasts is also accompanied by a lower expression level of Pax7 (Fig 4G and 4H) and higher expression levels of myogenic differentiation markers, e.g. *MyoG* and *Myh3* (Fig 4I). Thus, these results confirmed the *trans*-acting effects of DLL1 on Notch and myogenic gene expression.

Studies of *cis*-inhibition of Notch by their ligands have historically based on overexpression strategy [53]. We wish to examine the *cis*-inhibitory effect of DLL1 at its endogenous expression level by comparing WT or *DLL1*^KO^ myoblasts. To achieve this goal, we co-cultured human WT or *DLL1*^KO^ myoblasts with Dll1+ fibroblasts which serve as signal senders. Indeed, the expression levels of Notch target genes *HEY1* and *HEYL* in *DLL1*^KO^ myoblasts were significantly higher than those in WT myoblasts after mixing with fibroblasts (Fig 4J). The higher level of Notch is associated with drastic reduction of myogenic potentials of *DLL1*^KO^ myoblasts (Fig 4K and 4L). These results validate the cell-autonomous inhibition of Notch by DLL1 which is essential for the proper induction of myogenic differentiation.

Our results thus far suggest that only the signaling between Dll1– receiver and Dll1+ sender cell is effective, whereas the signaling among the Dll1+ sender cells is less fruitful. Reinforcing the signaling directionality, we show that Notch activation, recapitulated by direct expression of NICD, down-regulated DLL1 expression by 87% in human myocytes, together with drastic inhibitions of myogenic differentiation and markers’ expression (S1 Fig). Therefore, the Notch effector NICD and ligand Dll1 can reciprocally inhibit the other’s abundance, thus establishing Notch polarity in human myoblasts.

### *Cis*-inhibitory role of Dll1 is mediated by its extracellular domain

**Fig 5:**
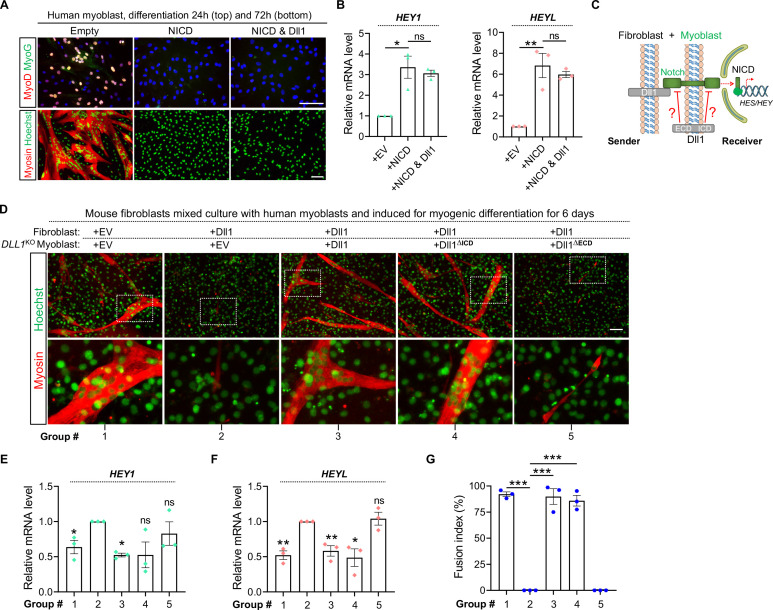
The extracellular domain of Dll1 is required for the *cis*-inhibition of Notch. (**A**) Immunostaining results of human myoblasts with retroviral expression of NICD alone or together with Dll1. Scale bar, 100 μm. (**B**) qPCR results of human myoblasts with retroviral expression of NICD alone or together with Dll1. Cells were differentiated for 24 hours. *n* = 3. (**C**) Schematic of Notch signaling and cell mixing experiment. (**D–F**) Myosin immunostaining (D) and qPCR (E, F) results of the co-cultured myoblast-fibroblast cells with expression of full length or mutated Dll1 proteins. EV, empty vector control. Note that deletion of extracellular domain abolishes the *cis*-inhibitory function of Dll1. (**G**) Measurement of myoblast fusion as shown in panel D. Treatments in groups 1–5 are same for panels D–G. Data are means ± SEM. *P < 0.05, **P < 0.01, ***P < 0.001. ns, not significant.

Following ligand-receptor interaction, Notch activity can be refined at multiple steps along the signal transduction pathway [14]. To probe the stage where Dll1 exerts *cis*-inhibitory effect on Notch, we expressed NICD alone or together with Dll1 in human myoblasts. Recapitulating the effects of Notch activation, expression of NICD significantly induced the expression of Notch targets and blocked the expression of MyoD/MyoG and myogenic differentiation (Fig 5A and 5B). Of note, co-expression of Dll1 failed to mitigate these effects from NICD (Fig 5A and 5B). Therefore, Dll1 may *cis*-inhibit Notch at upstream of the generation of NICD.

Previous structural studies of Notch ligand-receptor interactions suggest that both the *trans*- and *cis*-effects of ligand are carried by its extracellular domain through binding with Notch receptor [53, 54]. To validate this model, we employed *DLL1*^KO^ myoblasts to examine the *cis*-inhibition function of mutated Dll1 proteins where either the extracellular or intracellular domain was removed (Fig 5C). As a validation of our experiment design, we show that overexpression of Dll1 in fibroblasts (Fig 5D–5G, groups 2 vs 1) can consistently upregulate expression of Notch target genes in myoblasts and blocks their myogenic differentiation and fusion; such changes can be abolished when full-length Dll1 is re-expressed in *DLL1*^KO^ myoblasts (Fig 5D–5G, groups 3 vs 2). In this context, deletion of the extracellular domain but not intracellular domain of Dll1 protein abolished the *cis*-regulatory function of Dll1 (Fig 5D–5G, groups 4/5 vs 3). Therefore, consistent with previous biochemical studies in non-muscle cells, Dll1 in myoblasts also exerts the *cis*-regulatory function through the action of its extracellular domain.

### MyoD is the key regulator of Dll1 expression in human and mouse myoblasts

Given the significant roles of Dll1 in human myoblasts, we continued to dissect the transcriptional mechanism that governs its expression. The robust inductions of Dll1 during myogenic differentiation suggest that myogenic regulators, e.g., MyoD and MyoG, may directly govern Dll1 transcription. Supporting this notion, co-expression of MyoD or MyoG can restore Dll1 expression (S1C Fig) together with the myogenic differentiation of human myoblasts (S1D Fig) that were blocked by NICD expression (S1D Fig).

**Fig 6:**
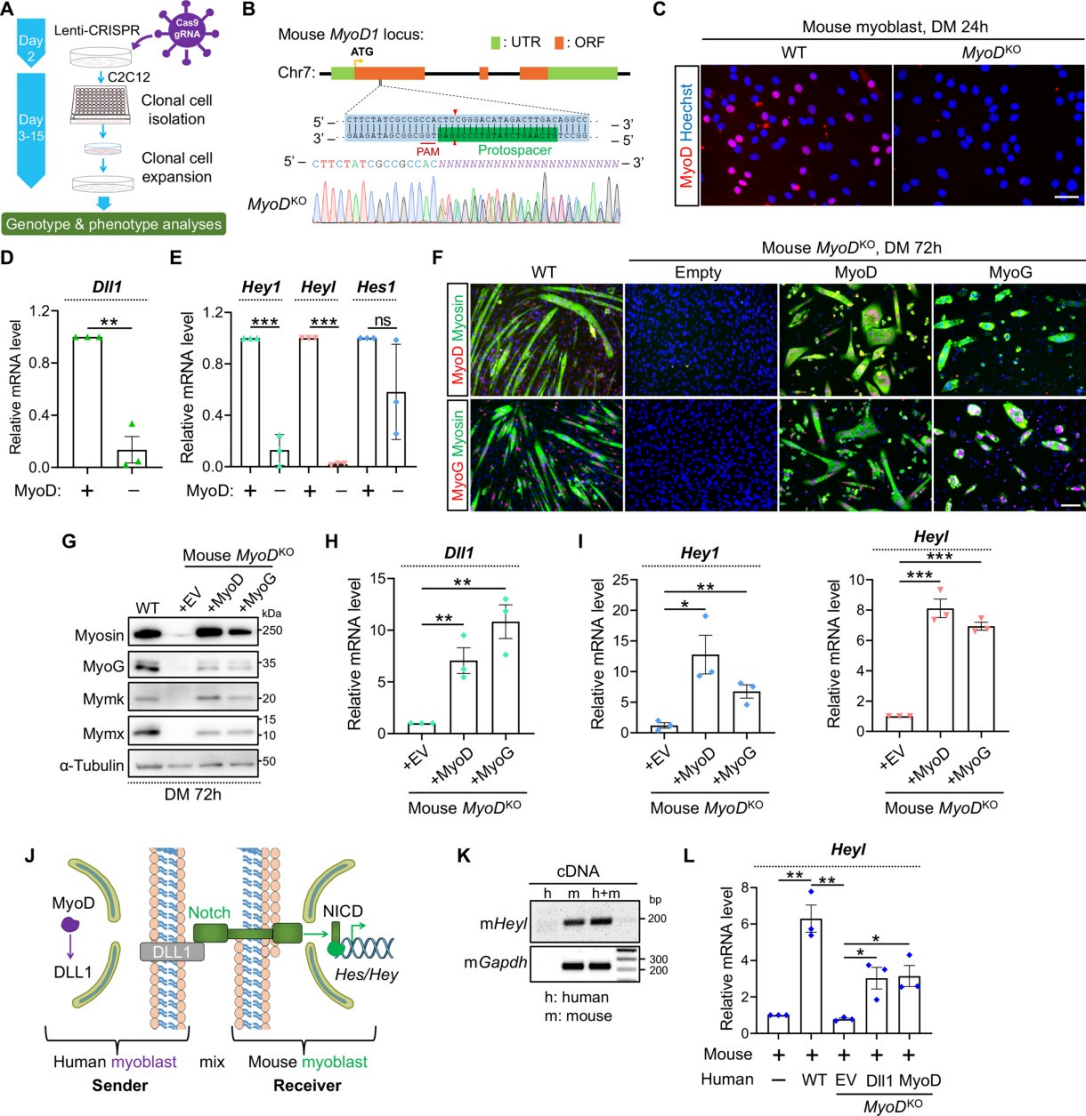
MyoD is essential for *Dll1* expression in mouse myoblasts. (**A**) Schematic of CRISPR/Cas9 approach to generate gene-knockout clones from mouse C2C12 myoblasts. (**B**) Sanger sequencing results of mouse *MyoD* genotyping PCR products. (**C**) Myod immunostaining confirmed depletions of MyoD protein in *MyoD*^KO^ clones. Scale bar, 50 μm. (**D**, **E**) qPCR results of *Dll1* (D) and Notch target genes (E) in WT and *MyoD*^KO^ myoblasts. Cells were differentiated for 48 hours. *n* = 3. (**F**) Immunostaining results of MyoD (top) or MyoG (bottom) together with myosin. Note that *MyoD*^KO^ myoblasts failed to differentiate and such a defect was rescued by retroviral expression of MyoD or MyoG. Cells were differentiated for 72 hours. Scale bar, 100 μm. (**G**) Western blotting results to show the expression levels of muscle proteins in WT or *MyoD*^KO^ myoblasts. Cells were differentiated for 72 hours. (**H**, **I**) qPCR results of *Dll1* (H) and Notch target genes (I) in mouse *MyoD*^KO^ cells. Myoblasts were differentiated for 72 hours. *n* = 3. (**J**) Schematic of Notch signaling that depicted the rationale of human and mouse myoblast-mixing assays. (**K**) Gel electrophoresis result that validated the specificity of mouse qPCR primers. (**L**) qPCR results that measured mRNA level of mouse *Heyl* using primers validated in K. Before mixing with mouse myoblasts, WT or *MyoD*^KO^ Human myoblasts were infected by retroviruses expressing Dll1 or MyoD. Cells were differentiated for 24 hours. *n* = 3. Data are means ± SEM. *P < 0.05, **P < 0.01, ***P < 0.001. ns, not significant.

To directly examine the regulatory relationship, we generated both mouse and human *MyoD*^KO^ myoblasts following a CRISPR–Cas9 workflow (Fig 6A). For mouse *MyoD* gene, one gRNA from the first coding exon was selected for the targeting. Following clonal expansion, genotyping analysis by sequencing revealed disruptions of MyoD ORFs (Fig 6B). As a result, MyoD protein was depleted from *MyoD*^KO^ myoblasts shown by immunostaining (Fig 6C). Notably, inactivation of MyoD significantly downregulated *Dll1* expression (Fig 6D), accompanied by drastic reductions of *Hey1* and *Heyl* expression (Fig 6E). In comparison, expression of *Hes1* showed a trend of reduction yet was not statistically significant compared with control group (Fig 6E).

When induced for myogenic differentiation, mouse *MyoD*^KO^ group did not to show any myosin+ cells (Fig 6F). Consistently, expression of myogenin (MyoG), myoblast-fusion factors myomixer (Mymx) and myomaker (Mymk) [55, 56] was undetectable in *MyoD*^KO^ cells (Fig 6G). To ascertain that such phenotypes were attributed to the exact loss of MyoD but not rare CRISPR off–target effect (if any), we performed rescue experiments. Indeed, myogenic differentiation (Fig 6F and 6G), expression of *Dll1* (Fig 6H) and Notch target genes (Fig 6I) were all restored when MyoD was re-introduced into *MyoD*^KO^ cells. Interestingly, forced expression of MyoG also achieved similar rescue effects (Figs 6F–6I).

In parallel, we examined the conservation of this regulatory mechanism in human cells. As such, we generated human *MyoD*^KO^ myoblasts by CRISPR mutagenesis using a pair of gRNA validated in our previous study [49]. Sequencing confirmed the biallelic frame-shift mutations in CRISPR treated cells (S2A Fig). Absence of MyoD protein was again validated by immunostaining (S2B Fig). Similar with the mouse data, deletion of MyoD from human myoblasts also abolished myogenic differentiation (S2B and S2C Fig) and significantly inhibited the expression of *DLL1* (S2D Fig) and *HEY1* (S2E Fig), which were collectively normalized upon re-expression of MyoD or MyoG protein in human *MyoD*^KO^ cells (S2B–S2E Fig).

To model the regulation of Notch activity by MyoD-Dll1 axis, we performed heterologous cell-mixing experiment. In this assay, WT or *MyoD*^KO^ human myoblasts were mixed with mouse myoblasts (Fig 6J). As such, the *trans*-acting effect upon disruption of MyoD can be examined by comparing Notch target gene expression between various cell-mixing groups. We first validated the mouse sequence-specificity of qPCR primers (Fig 6K). Of note, after mixing with WT-human myoblasts, *Heyl* expression was significantly induced in mouse myoblasts (Fig 6L). However, this effect was abolished when human *MyoD*^KO^ myoblasts were used for the mixing experiment (Fig 6L). Such deficiency of *Heyl* expression was rescued when Dll1 or MyoD was provided back to human *MyoD*^KO^ myoblasts (Fig 6L). These results suggest that MyoD, by upregulation of Dll1, can transactivate Notch signaling in adjacent myoblasts.

### Disparities of Dll1 expression changes upon genetic deletions of MyoG from human and mouse myoblasts

MyoG is a direct target gene of MyoD during myogenic differentiation [57, 58]. Of note, similar with *Dll1*, expression of MyoG was also abolished in both mouse (Fig 6F and 6G) and human (S2C Fig) *MyoD*^KO^ cells. Intriguingly, the biological deficiency of *MyoD*^KO^ cells can be rescued by forced expression of MyoG, indicating that MyoD may control Dll1 expression through MyoG.

**Fig 7:**
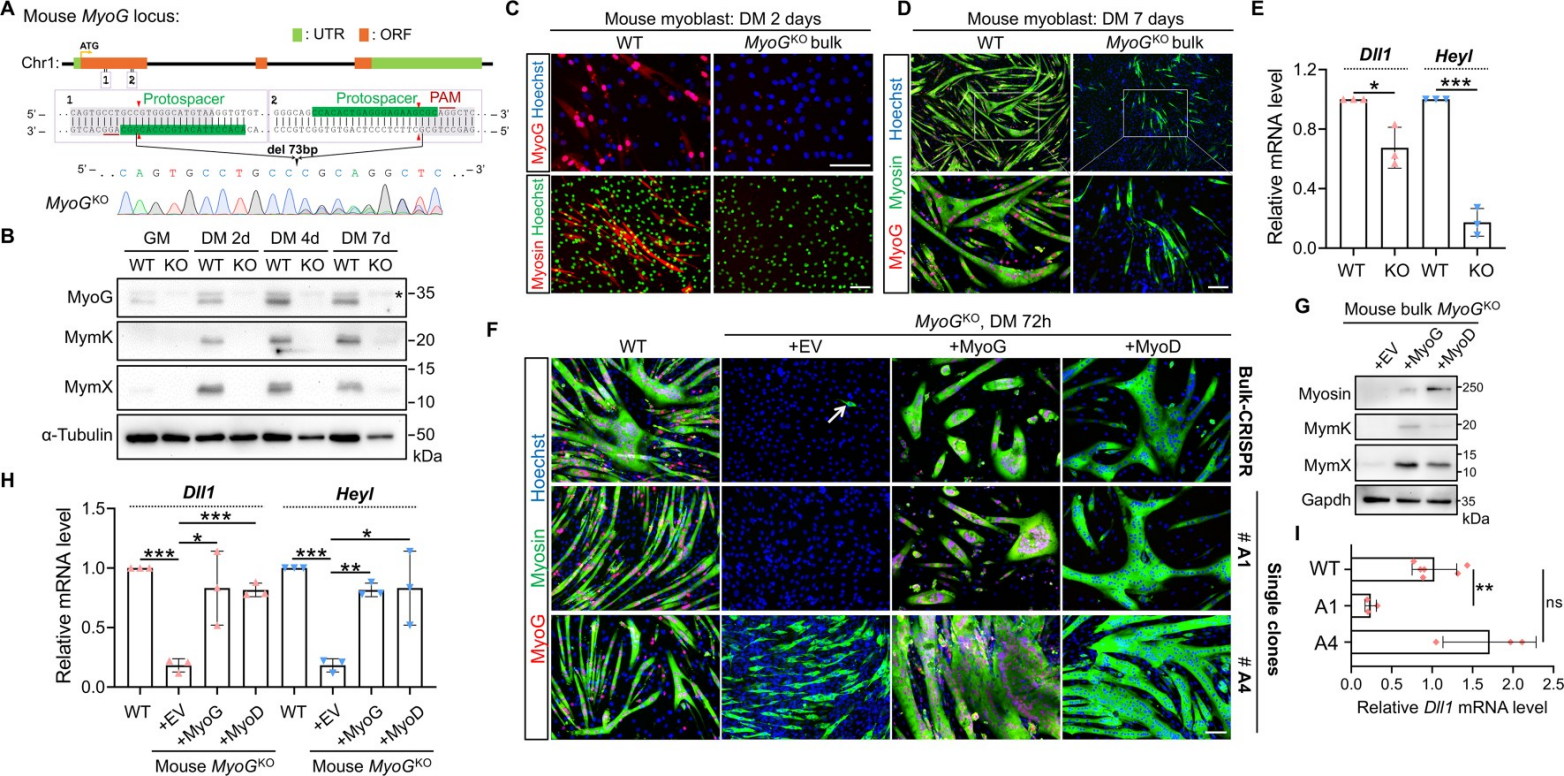
Responses of *Dll1* expression upon deletion of *MyoG* from mouse myoblasts. (**A**) Schematic of mouse *MyoG* gene structure and an example of Sanger sequencing result after CRISPR/Cas9 mediated gene editing in C2C12 mouse myoblasts. (**B**) Western blotting results to show the expression levels of myogenic markers in WT and *MyoG*^KO^ myoblasts. Star highlights a non-specific band. (**C**, **D**) Immunostaining results of mouse WT and *MyoG*^KO^ myoblasts at day 2 (C) and day 7 (D) post differentiation. (**E**) qPCR results of *Dll1* and *Heyl* in WT and *MyoG*^KO^ myoblasts. Cells were differentiated for 48 hours. *n* = 3. (**F**) Immunostaining result of MyoG and myosin for bulk CRISPR-treated myoblasts (top) and two isolated KO clones (bottom). Note that although MyoG were invariably depleted, the two KO clones showed a large variation of myogenic capacity. Cells were induced for differentiated for 72 hours. (**G**) Western blotting results to show the expression levels of myogenic differentiation markers in and bulk CRISPR-treated *MyoG*^KO^ myoblasts. Cells were differentiated for 72 hours. (**H**, **I**) qPCR results for gene expression in WT and bulk CRISPR-treated *MyoG*^KO^ myoblasts (H) or *MyoG*^KO^ single-knockout clones (I). Cells were differentiated for 48 hours. *n* = 3. Data are means ± SEM. *P < 0.05, **P < 0.01, ***P < 0.001. ns, not significant. Scale bars, 100 μm.

To directly test a role of MyoG in MyoD–Dll1 regulatory axis, we inactivated *MyoG* gene through CRISPR mutagenesis in mouse (Fig 7A) and human myoblasts (S3A Fig) [49]. Successful depletions of MyoG proteins were confirmed by Western blotting (Fig 7B) and immunostaining (Figs 7C and S3B) using an antibody that detects the carboxyl terminus of myogenin. Given the high efficiency of CRISPR editing in C2C12 myoblasts (Fig 7B and 7C), we first characterized the bulk CRISPR-treated cells before isolating single-clone mutants. Of note, the majority of *MyoG*^KO^ C2C12 myoblasts did not express myosin after full-term myogenic differentiation (Fig 7D). In alignment with the fusogenic defects, expression of Mymx and Mymk genes was abolished in mouse *MyoG*^KO^ cells (Fig 7B). For *Dll1* expression however, we only observed a mild though significant reduction (33% lower) in mouse *MyoG*^KO^ cells (Fig 7E). In comparison, the reductions for expression of the Notch target gene *Heyl* were more pronounced (83% lower) (Fig 7E). Validating the specific effect of CRISPR-knockout, myoblast differentiation (Fig 7F, top row) as well as gene expression changes (Figs 7G and 7H) were rescued upon re-expression of MyoG. More interestingly, forced expression of MyoD can also rescue the phenotypes of *MyoG*^KO^ cells, suggesting that MyoD can regulate Notch and myogenic gene expression independent of MyoG.

In contrast to the complete myogenic failure of *MyoD*^KO^ myoblasts, myosin+ cells were spotted from the bulk CRISPR-treated *MyoG*^KO^ myoblasts (Fig 7F). We postulated that this may reflect a heterogeneity of C2C12 myoblasts [59] which showed various responses upon MyoG deletion, or simply due to the lack of uniformity of gene knockout. Thus, we set out to isolate and characterize the *MyoG*^KO^ single-clones. Indeed, while the majority of *MyoG*^KO^ clones showed the complete failure of differentiation (e.g. A1 clone), myosin+ cells were readily detected among a few clones after myogenic induction (e.g. A4 clone) (Fig 7F). Interestingly, the changes of Dll1 expression corelated well with myogenic potentials of *MyoG*^KO^ clones (Fig 7I). Similarly, human *MyoG*^KO^ myoblasts, which retain myogenic potential (myosin+) despite of a fusion defect (S3B and S3C Fig), showed a relatively normal level of expression for *DLL1*, compared with control group (S3D Fig).

To rule out an effect of cell immortalization on the gene expression changes above, we also repeated MyoD/MyoG CRISPR experiments in primary mouse myoblasts. Because these cells are not amendable for clonal expansion and characterization, we opted to directly examine the expression of *Dll1* gene in the bulk CRISPR-treated cells (S4A Fig). Reflecting high efficiency of knockout, we observed a drastic reduction of MyoD or MyoG expression in the CRISPR treated groups (S4B Fig). Of note, inactivation of MyoD also reduced the expression of MyoG, while deletion of MyoG has no obvious effect on the expression level of MyoD (S4B Fig), consistent with the regulation relationship between these factors. Nonetheless, both MyoD and MyoG CRISPR treatments caused major defects of myogenic differentiation (S4B Fig), accompanied by significant reductions of Dll1 expression (S4C Fig). Together, our analyses of mouse and human gene knockout myoblasts suggest that the induction of *Dll1* expression is an integral part of myogenic program for muscle cells under the control by MyoD and MyoG.

### MyoD induces Dll1 transcription in non-muscle cells

Our gain- and loss-of-function experiments unveiled the crucial roles of MyoD in controlling Dll1 expression in both human and mouse myoblasts. We continued to study the mechanistic basis underlying this regulation and focused on two questions. First, is the transactivator MyoD sufficient to induce Dll1 expression? Second, how MyoD transactivates Dll1 expression?

**Fig 8:**
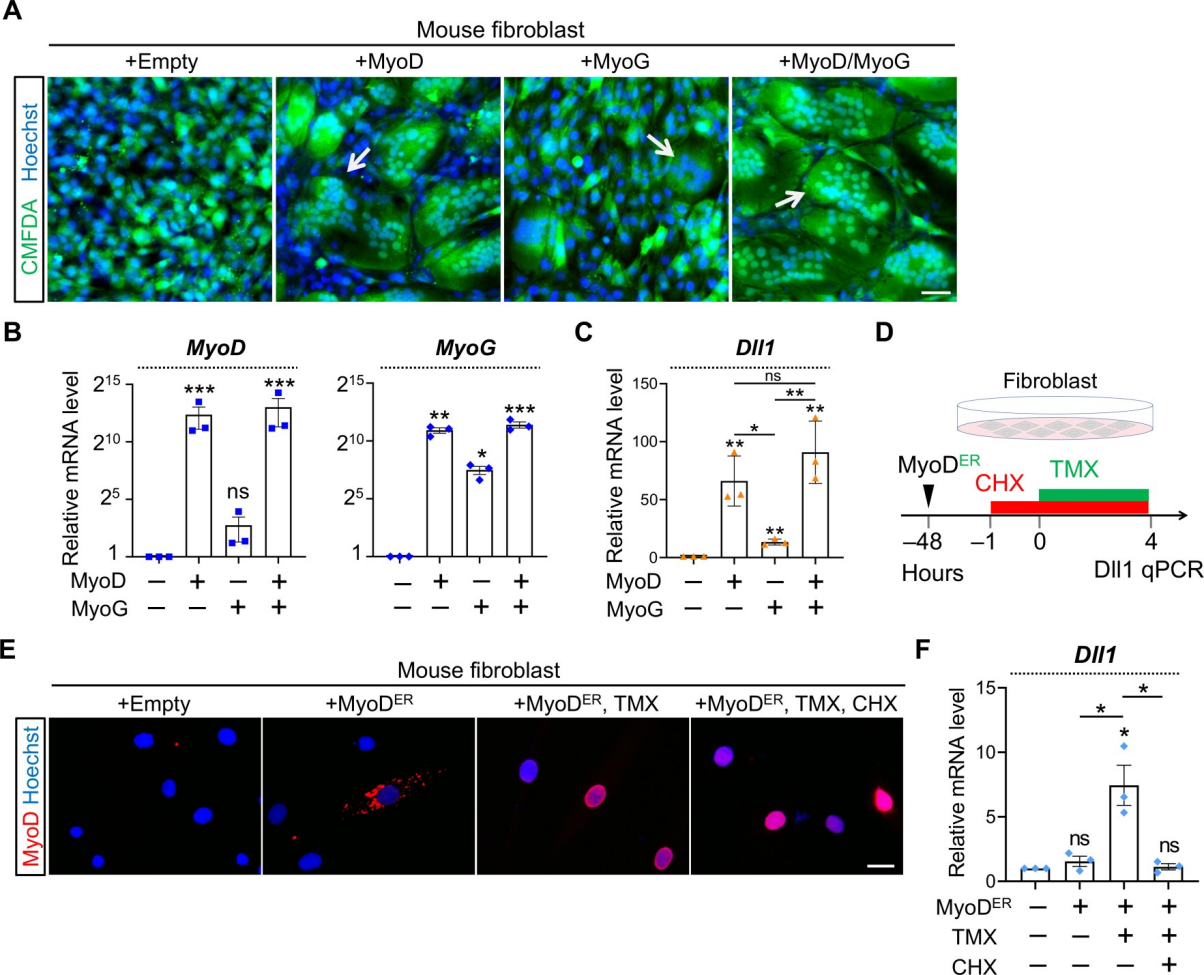
Sufficiency test of *Dll1* inductions by MyoD in fibroblasts. (**A**) Fluorescence images of cell cytosol dye CMFDA (green) to highlight fibroblast syncytia induced by MyoD or MyoG. Scale bar, 100 μm. (**B**, **C**) qPCR results of *MyoD*, *MyoG* (B), and *Dll1* in mouse fibroblasts with forced expression of MyoD, MyoG or both. *n* = 3. (**D**) Schematic of experimental design to test the sufficiency of MyoD transcriptional activity in activating *Dll1* expression in fibroblasts. (**E**) MyoD immunostaining of fibroblasts. TMX: tamoxifen. CHX: cycloheximide. Scale bar, 25 μm. (**F**) qPCR results of *Dll1* expression in fibroblasts after treatment illustrated in D. Note that induction of *Dll1* expression by MyoD was abolished upon CHX treatment (5 hours). *n* = 3. Data are means ± SEM. *P < 0.05, **P < 0.01, ***P < 0.001. ns, not significant.

We investigated the first question by performing sufficiency test in fibroblasts which do not express any myogenic factor. We transduced fibroblasts with MyoD or (and) MyoG. Consistent with our previous report that MyoD is sufficient to induce the expression of Mymx and Mymk [49], syncytia of fibroblasts are produced (Fig 8A). Strikingly, expression of MyoD in fibroblast robustly activated Dll1 expression by 66 folds (Fig 8B and 8C). MyoG also induced Dll1 expression though at much weaker levels compared with MyoD (Fig 8C). Again, expression of MyoG was also strongly upregulated by MyoD (Fig 8B, right) whereas MyoG does not significantly induce expression of MyoD (Fig 8B, left). Consistent with the dominant effect of MyoD, the inductions of Dll1 expression by MyoD was not significantly boosted by adding MyoG (Fig 8C).

During the fate conversion of fibroblasts, MyoD induces a pan-myogenic program exemplified by the upregulation of MyoG expression (Fig 8B, right) [60–63]. We continued to examine whether MyoD requires other myogenic factors at its downstream to induce Dll1 expression. To test this, we concomitantly inhibited protein translation by cycloheximide (CHX) with the control of MyoD transcriptional activities (Fig 8D). The later was achieved through commanding nuclear importing of a MyoD-estrogen receptor fusion protein (MyoD^ER^) [64] with treatment of 4-hydroxytamoxifen (TMX). We reasoned that if MyoD protein is self-sufficient to activate Dll1 expression, such an induction should not be negated by CHX treatment which blocks translations of other myogenic factors, e.g. MyoG; by contrary, if CHX compromises the action of MyoD, it would suggest that MyoD requires function from other factor(s) to cooperatively activate Dll1 expression.

This experiment design was validated previously [49] and also confirmed here by showing the nuclear import of MyoD^ER^ protein in response to TMX treatment (Fig 8E). Notably, activation of MyoD^ER^ robustly induced Dll1 transcription (Fig 8F). Such an effect was completely abolished when CHX was administered together with TMX (Fig 8F). Therefore, additional factor(s), which can be induced by MyoD, is required by MyoD to activate Dll1 transcription. This result is in contrast with the report that MyoD is self-sufficient to induce Dll1 expression during *Xenopus* embryogenesis [31]. As such, mammals may have evolved an extra layer of regulatory mechanism to fine-tune Dll1 expression during myogenesis.

### Perturbations of *cis*-regulatory motifs on Dll1 promoter affect its expression *in vivo* and *in vitro*

**Fig 9:**
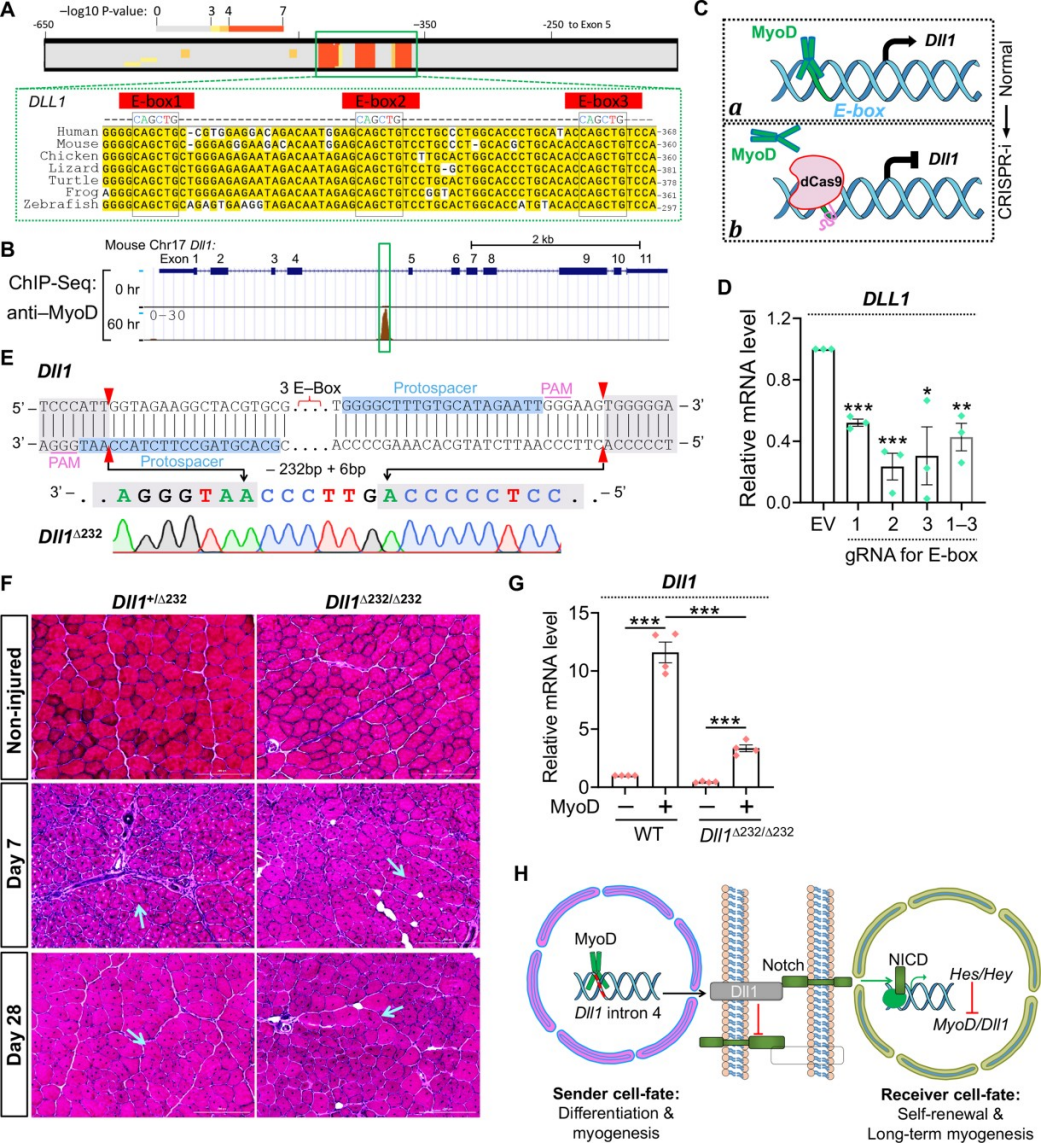
Regulation of *Dll1* expression by MyoD through intronic E-box elements in human and mouse cells. (**A**) Bioinformatic predictions of MyoD binding motifs in the *Dll1* intronic regions from distantly related mammalian species. (**B**) ENCODE ChIP-seq results of MyoD from mouse myoblasts. Green box highlights the mouse sequence displayed in A. (**C**) Schematic of experiment design and rationale to probe *cis-*regulatory elements by dCas9 mediated interference. CRISPRi: CRIPSR interference; (**D**) qPCR results of human WT myoblasts with expression of dCas9 and gRNA indicated. *n* = 3. (**E**) Sequencing result of mouse *Dll1*^Δ232^ mutant allele generated by CRISPR/Cas9 mediated gene targeting. (**F**) H&E staining results of cross-sections from control and injured tibialis anterior muscles. Arrows point to regenerated myofibers with centronuclei. Scale bar, 200 μm. (**G**) qPCR results that measured *Dll1* expression in mouse fibroblasts that infected by control or MyoD-expressing retroviruses. *n* = 4. (**H**) Schematic summary of transcriptional regulations and ligand-receptor interactions between Notch signal sending or receiving cells. Data are means ± SEM. *P < 0.05, **P < 0.01, ***P < 0.001.

As a bHLH transcriptional factor, MyoD activates the expression of its target genes through binding to E-box motifs (*CANNTG*) [62]. Using FIMO, a motif discovery tool that empirically predicts transcriptional factor binding sites [65], we discovered three highly conserved MyoD-binding motifs in the intron 4 of Dll1 gene (Fig 9A). Of note, analysis of an ENCODE ChIP-seq dataset [66] also discovered a MyoD-binding peak in this region during C2C12 myoblast differentiation (Fig 9B).

We then utilized a CRISPR interreference tool to interrogate gene regulatory mechanism as previously reported [49, 67–69]. For this experiment, a catalytically dead Cas9 (dCas9) was guided to these E-box motifs to dissect their function underlying the MyoD–Dll1 regulatory axis (Fig 9C). We hypothesized that MyoD can bind to the intronic E-box motifs to induce Dll1 expression during myogenic differentiation (Fig 9C, state ***a***); when recruited by gRNA, the positioning of dCas9 to these E-box motifs should block MyoD binding thus repressing the expression of Dll1 (Fig 9C, state ***b***). Consistent with our hypothesis, expression of gRNA that recruits dCas9 to each of these three E-box motifs significantly inhibited Dll1 expression by an average of 65% in human myoblasts (Fig 9D). No additive effect was observed when these E-boxes were simultaneously blocked (Fig 9D). Therefore, MyoD can activate Dll1 expression in human myoblasts at least partially through binding to the evolutionarily conserved E-box motifs located in the intron 4 of Dll1 gene.

The CRISPR interference results prompted us to test the function of these *cis*-regulatory elements during myogenesis *in vivo*. Using CRISPR genome editing, we generated a novel mouse model that deleted these E box motifs from intron 4 of Dll1 gene. Sequencing confirmed the removal of a 232 bp region centered on these conserved E-box elements (Figs 9E and S5A). We named this allele as *Dll1*^Δ232^. Germline transmission of *Dll1*^Δ232^ allele was confirmed after breeding the founder with WT mice. Intercrosses of *Dll1*^Δ232/+^ mice produced homozygous mutants at expected Mendelian ratios (S5B Fig). *Dll1*^Δ232/Δ232^ mutants were overly normal and fertile, which are in stark contrast to the premature lethality that was observed for *Dll1* null mutants [37].

Given Dll1 expression was strongly induced by muscle injury (Fig 1E), we first tested whether deletion of the intronic E-box elements can affect Dll1 expression and muscle regeneration. Surprisingly, in contrast to major defect of muscle regeneration in Dll1 gene knockout model [70], normal progressions of muscle regeneration at day–4, –7 and –28 post injury were observed for *Dll1*^Δ232/Δ232^ mutant mice (Figs 9F and S5C and S5D). To better interpret this result, we examined expression of Dll1. Fibroblasts were isolated from control and mutant mice followed by overexpression of MyoD. Interestingly, the Dll1 expression in mutant fibroblasts showed a 71% decrease compared with control group (Fig 9G). This effect is similar with that achieved by CRISPR interference (65% lower) in human myoblasts (Fig 9D). In reviewing the ChIP-seq data [66], we did not observe additional MyoD binding peak in other introns or intergenic regions. Lastly, a recent study using luciferase assay also identified this intronic region as MyoD binding site [70]. Therefore, we reasoned that *Dll1*^Δ232/Δ232^ mutant cells may have used alternative E-box motifs from distal enhancer of an unknown region to compensate the knockout or CRISPR-interference effects *in vivo*.

## Discussion

In summary, our study uncovered the crucial regulations of Dll1 expression and Notch activation by myogenic factor MyoD. This intercellular regulatory loop (Fig 9H) involves three major parts that could be initiated by asymmetric divisions of muscle precursor cells [71–73] that allows one daughter cell to inherit a higher level of MyoD protein than the other. First, the MyoD^high^ myoblast can promptly turn on the expression of Dll1 which transactivates Notch in adjacent MyoD^low^ cell. Second, activation of Notch in MyoD^low^ cell will further downregulate transcriptions of MyoD and Dll1 genes. Third, even in presence of any residual expression of Dll1 from MyoD^low^ cell, binding of these ligand with Notch receptor in MyoD^high^ cells will unlikely activate Notch in the latter due to *cis*-inhibition from the abundantly expressed Dll1. Similarly, Notch signaling between MyoD^high^ cells will also be inhibited. Collectively, the polarities of Notch can be established that allow daughter cells to adapt opposite cell-fates, i.e. differentiation or self-renewal. Specifically, prompt upregulation of Dll1 in differentiating cells can refrain cell-autonomous Notch activity. Given the strong inhibitory effects of Notch on myogenesis, such *cis*-inhibitory mechanism is a prerequisite for completion of myogenic differentiation. On the other side, transactivation of Notch pathway may prevent precocious differentiation and promote a quiescence state and the self-renewal of muscle stem cells for long-term muscle regeneration (Fig 9H). Together with previous studies [27, 35], these results enabled a more complete understanding of the feedback mechanism whereby Notch polarity and the divergent cell-fates can be established during myogenesis.

Notch activity is sensitive to the molar ratios of ligand and receptor. The *cis*-inhibitory effect of ligand on Notch was initially observed in the developmental processes of *Drosophila* [53, 74]. Reflecting the complexity of mammalian system, *cis*-interaction of receptor with ligand was shown to either inhibit or activate Notch in different biological processes [26, 75, 76]. Nonetheless, our study provides the direct evidence that Dll1 can attenuate Notch signaling cell-autonomously to facilitate myogenic differentiation of myoblasts. Our structure and function analysis of Dll1 revealed the key roles of its extracellular domain for the *cis*-inhibition. This result is consistent with previous structural determination of the Notch ligand/receptor protein complexes [54]. The *cis* interaction of ligand with receptor may either titrate receptor and limit it from *trans*-interacting with ligand from sender cells [53] or the ligand *in cis* can stabilize Notch receptor such that the proteolytic processing and activation of receptors are interfered [53].

Another interesting question that can be explored is the role of Jag1 in this model. During myogenic differentiation of mouse and human myoblasts, expression of Jag1 was also induced, though not as strongly as Dll1. Of note, previous study showed that Jag1 is the least-efficient ligand in activating Notch in myoblast [77]. Therefore, the upregulation of Jag1 expression during myoblast differentiation may have smaller impact on the Notch activity in adjacent cells, compared with that from Dll1.

In addition to the gene gain- and loss-of-function experiments, we also examined the *cis*-regulatory elements underlying the control of Dll1 transcription through two complementary approaches: CRISPR-interference in human and CRISPR-knockout in mouse. Results from these experiments consistently revealed the crucial role of the intronic E-box motifs in driving Dll1 expression. As such, the interference and knockout of these elements produced 65% and 71% reductions of Dll1 expression, respectively. However, this magnitude of change failed to elicit any obvious phenotype *in vivo* during muscle development or regeneration. A few possibilities may explain this result. First, downregulation of Dll1 may triggered upregulation of other Notch ligands in muscle cells that compensated for the reduction of Dll1. Second, a residual 29% of Dll1 expression in mutant myoblasts could be sufficient to elicit *cis*-inhibitory and *trans*-activating effects such that the Notch activity and cell fate are not impacted. Nevertheless, the genetic mouse model that we generated could be applied to understand whether these *cis*-regulatory sites can be utilized by other bHLH transcriptional factors, e.g., NeuroD [78], during other developmental processes or diseases. In addition, future efforts are warranted to comprehensively dissect the unknown *cis*-regulatory sequence whereby MyoD drives Dll1 expression during myogenic differentiation. Lastly, the mysterious factor(s) at downstream of MyoD that cooperatively induces Dll1 expression also needs to be identified. Although MyoG appeared as a tempting candidate, the observation that MyoD can robustly induce Dll1 expression from *MyoG*^KO^ cells indicates that other factor(s) must exist in regulation of this process.

## Materials and methods

### Generation of *Dll1*^Δ232^ mutant mice

All animal procedures were approved by the *Institutional Animal Care and Use Committee* (IACUC) at the University of Georgia. *Dll1*^Δ232^ mouse model was generated by oviduct electroporation as previously reported [79]. Briefly, the copulated female mice were used for surgery to exposes oviduct. CRISPR gene editing cocktails were freshly assembled and contained 6 μM Cas9 protein (IDT, 1081058, Lot # 0000405530), 30 μM gRNA (crRNA annealed with tracrRNA, IDT, 1072534, Lot # 0000403961). This cocktail was delivered into oviduct through microcapillary injection. Oviduct electroporation was performed using CUY21EDIT II electroporator with the following protocol: Pd A: 100 mA, Pd on: 5 ms, Pd off: 50 ms, three cycles, decay 10%. Muscle injury was induced by injecting 1.2% barium chloride (50 μl) into tibialis anterior muscle.

### Mouse genotyping analysis

Dll1 genotyping PCR was performed using genomic DNA extracted from the toe clipping with the primers, forward: AGAACCTCTGTTCGTGCCTG and reverse: GCGTCTAGGACAAAAGGGCT. For Sanger sequencing, PCR products were first gel purified and cloned into pCRII Topo vector (Thermo Fisher Scientific, K460001) and sequenced with T7 or SP6 primers. Germline transmission of the *Dll1*^Δ232^ allele was confirmed by genotyping *F1* generation mice.

### Cell cultures

Human myoblasts (ID: hSkMC-AB1190) were immortalized as previously described [48]. Human myoblasts were cultured in Skeletal Muscle Cell Growth Medium (PromoCell, C-23060). Mouse 10T1/2 fibroblasts (ATCC, CCL-226) and C2C12 myoblasts (ATCC, CRL-1772) were maintained in 10% FBS with 1% penicillin/streptomycin (Gibco, 15140122) in DMEM (Dulbecco’s Modified Eagle’s Medium-high glucose, D5796). Primarily myoblasts were isolated by enzyme digestion following the protocol [80]. Human and mouse myoblast differentiation medium contains 2% horse serum supplemented in DMEM with 1% antibiotics penicillin/streptomycin. Cells were tested mycoplasma negative using Universal Mycoplasma Detection Kit (ATCC, 30-1012K).

### Lentivirus preparation and CRISPR-Cas9 knockout experiments *in vitro*

Lenti-CRISPR v2 vector [81] used for gene knockout experiments *in vitro* was a gift from Feng Zhang (Addgene plasmid # 52961). The guide RNAs that target the coding regions of human and mouse MyoD and MyoG genes were individually cloned into the Lenti-CRISPR v2 vector and sequenced to verify the correct insert. The sequences for gRNA used in this study are provided.

Mouse Dll1 intron 4 gRNA1: GCACGTAGCCTTCTACCAAT

Mouse Dll1 intron 4 gRNA2: GGGGCTTTGTGCATAGAATT

Mouse MyoD gene: GTCAAGTCTATGTCCCGGAG

Mouse MyoG gene gRNA1: ACACCTTACATGCCCACGGC

Mouse MyoG gene gRNA2: CCACACTGAGGGAGAAGCGC

Human MyoD gene: CGTCGAGCAATCCAAACCAG

Human MyoG gene gRNA1: ACCACCAGGCTACGAGCGGA

Human MyoG gene gRNA2: CCACACTGAGGGAGAAGCGC

Human DLL1 gene gRNA1: ACCCAGAGGCACCTGACGGT

Human DLL1 gene gRNA2: TGACGAACACTACTACGGAG

Lentivirus was produced by transfecting Lenti-X 293T cells (Clontech, 632180) using FuGENE6 transfection reagent (Promega, #E2692) with pLenti-V2, psPAX2 and pMD2.G plasmids. 48 hours post transfection, lentivirus was collected to infect human or myoblast myoblasts. psPAX2 vector was a gift from Didier Trono (Addgene plasmid # 12260). pMD2.G vector was a gift from Didier Trono (Addgene plasmid # 12259).

### Retroviral vector preparations and expression

Retroviral expression vector pMXs-Puro (Cell Biolabs, # RTV-012) was used for gene cloning and expression in human and mouse myoblasts. The open reading frames for red fluorescent protein Cherry and mouse Dll1 were cloned into pMXs-Puro vector by In-Fusion cloning. The identities of the DNA inserts in the plasmids were Sanger sequencing verified. MyoD-pCLBabe plasmid [82] was a gift from Stephen Tapscott (Addgene plasmid # 20917). Myoblasts were labelled by retroviral infection that delivers the pMXs-Cherry expression vector. MyoDER expression and chemical treatments were performed as previously reported [49].

### Differentiation index and fusion index measurements

Differentiation index was calculated as the percentage of the nuclei number within muscle cells (MF20+) divided by the total nuclei number in the imaging area. Fusion index was calculated as the nuclei number in myotubes (≥ 3 nuclei) as a percentage of the total number of nuclei inside muscle cells.

### RNA extraction and gene expression analysis

RNA was extracted by using TRIzol Reagent (Thermo Fisher Scientific). Before used for reverse transcription, RNA quality and concentration were assessed by using a spectrophotometer (Nanodrop One, Thermo Fisher Scientific). cDNA was synthesized by reverse transcription using random oligos with M-MLV reverse transcriptase (Invitrogen, 28025013). To analyze gene expression changes, the Real-time PCR was performed using QuantStudio 3 Real-Time PCR System (Thermo Fisher Scientific) with SYBR Green Master Mix (Roche) and qPCR primers. The 2^ΔΔCt^ method was used to analyze gene expression after normalization to expression of Gapdh or 18S rRNA. Primer sequences used in this study are provided.

Primers for DLL1 qPCR-F: GATTCTCCTGATGACCTCGCA

Primers for DLL1 qPCR-R: TCCGTAGTAGTGTTCGTCACA

Primers for JAG1 qPCR-F: GTCCATGCAGAACGTGAACG

Primers for JAG1 qPCR-R: GCGGGACTGATACTCCTTGA

Primers for DLL3 qPCR-F: CACTCCCGGATGCACTCAAC

Primers for DLL3 qPCR-R: GATTCCAATCTACGGACGAGC

Primers for DLL4 qPCR-F: GTCTCCACGCCGGTATTGG

Primers for DLL4 qPCR-R: CAGGTGAAATTGAAGGGCAGT

Primers for JAG2 qPCR-F: TGGGCGGCAACTCCTTCTA

Primers for JAG2 qPCR-R: GCCTCCACGATGAGGGTAAA

Primers for HEY1 qPCR-F: AACTGTTGGTGGCCCTGAAT

Primers for HEY1 qPCR-R: CAATTGACCACTCGCACACC

Primers for HEYL qPCR-F: ATGAGTCCTGGGAGAGACCC

Primers for HEYL qPCR-R: GCCAGTCAGTCATTGCTCCT

Primers for HES1 qPCR-F: TTTTTGGCGGCTTCCAAGTG

Primers for HES1 qPCR-F: GGTGGGCTAGGGACTTTACG

Primers for MYOD1 qPCR-F: CGACGGCATGATGGACTACA

Primers for MYOD1 qPCR-R: TATATCGGGTTGGGGTTCGC

Primers for MYOG qPCR-F1: GCCAACCCAGGGGATCAT

Primers for MYOG qPCR-R1: CCCGGCTTGGAAGACAATCT

Primers for MYH3 qPCR-F: ATTGCTTCGTGGTGGACTCAA

Primers for MYH3 qPCR-R: GGCCATGTCTTCGATCCTGTC

Primers for GAPDH qPCR-F: CACCAGGTGGTCTCCTCTGA

Primers for GAPDH qPCR-R: CAAGGGGTCTACATGGCAACT

Primers for 18S qPCR-F: GTAACCCGTTGAACCCCATT

Primers for 18S qPCR-R: CCATCCAATCGGTAGTAGCG

Primers for Dll1 qPCR-F: CAGGACCTTCTTTCGCGTATG

Primers for Dll1 qPCR-R: AAGGGGAATCGGATGGGGTT

Primers for Hey1 qPCR-F: GCACGCCACTATGCTCAATG

Primers for Hey1 qPCR-R: GGGGACCTAGACTACCAGCA

Primers for Heyl qPCR-F: CCACTGGCGCAGATGAGTTA

Primers for Heyl qPCR-R: ATCCTGTTGGCTTGGGATGG

Primers for Hes1 qPCR-F: TTTTTGGCGGCTTCCAAGTG

Primers for Hes1 qPCR-R: GGTGGGCTAGGGACTTTACG

Primers for Gapdh qPCR-F: TCTCCTGCGACTTCAACAGC

Primers for Gapdh qPCR-R: AGTTGGGATAGGGCCTCTCTT

Primers for 18s qPCR-F: ACCGCAGCTAGGAATAATGGA

Primers for 18s qPCR-R: GCCTCAGTTCCGAAAACCA

### Western blotting

Protein lysate was prepared by using RIPA buffer (Sigma, R0278) supplemented with complete protease inhibitor (Sigma). Lysates were centrifuged at 16,000 x g for 15 minutes. The resultant supernatant was mixed with 4x Laemmli sample buffer (Bio-Rad, #161–0747). Total 20 μg protein lysates were separated by SDS-PAGE gel electrophoresis. The proteins were transferred to a polyvinylidene fluoride membrane and blocked in 5% milk at room temperature, and incubated with following primary antibodies diluted in 5% milk overnight at 4°C. Gapdh (Santa Cruz Biotechnology, sc-32233), α-Tubulin (Santa Cruz Biotechnology, sc-8035), myosin (DSHB, MF20), MyoD (Santa Cruz Biotechnology, SC-304), MyoG (DSHB, F5D). The HRP-conjugated secondary antibodies: Donkey anti-sheep IgG-HRP (Santa Cruz Biotechnology, sc-2473), Goat Anti-Mouse IgG (H+L)-HRP Conjugate (Bio-Rad, 170–6516) and Goat Anti-Rabbit IgG (H + L)-HRP Conjugate (Bio-Rad, 170–6515) were diluted at 1:5,000. Signal detection was performed using Western Blotting Luminol Reagent (Santa Cruz Biotechnology, sc2048).

### CRISPR interreference assays

Lenti-SAM v2 was a gift from Adam Karpf (Addgene plasmid # 92062). Lenti-SAM v2 plasmid was used for gRNA cloning after the removal of VP64 expression cassette. The gRNA sequences that target the control and E-box motif regions of human DLL1 intronic region were provide below.

Human DLL1 intron 4 gRNA1: CTGCCCCAGCGCAACAATGC

Human DLL1 intron 4 gRNA2: ATGGAGCAGCTGTCCTGCCC

Human DLL1 intron 4 gRNA3: AGAGTGGACAGCTGGTATGC

### Immunostaining

Immunostaining was performed as previously reported [49]. Briefly, cells were fixed in 4% PFA for 10 minutes, membrane was permeabilized using 0.2% Triton X-100. Cells are blocked with 3% BSA at room temperature. Primary antibody incubation was performed overnight at 4°C. Immunostaining signal was detected by incubating with fluorescence conjugated secondary antibodies and Hoechst 33342 to visualize nucleus. Fluorescence images were collected using the BioTek Microscope System or Olympus FV1200 Confocal Laser Scanning Microscope.

### Quantification and statistical analysis

Experiments were repeated at least three times. All quantitative results were analyzed with student’s *t* test with a two-tail distribution. Comparisons with *P* values < 0.05 were considered significant.

## Supporting information

S1 Fig Notch activation inhibits *DLL1* expression in human cells through MyoD/MyoG. (**A–C**) qPCR results of myogenic markers (A), *DLL1* (B, C) in human myoblasts with retroviral expression of NICD. Note that inhibitory effect of NICD on *DLL1* expression was rescued by MyoD or MyoG expression (C). Cells were differentiated for 72 hours. *n* = 3. Data are means ± SEM. **P < 0.01, ***P < 0.001. (**D**) MyoG and myosin immunostaining results of human myoblasts after differentiation for 72 hours. Note that myogenic differentiation and fusion defects of NICD can be rescued by co-expression of MyoD or MyoG. Scale bar, 100 μm.
(TIF)

S2 Fig MyoD is essential for *DLL1* expression in human myoblasts. (**A**) Human *MyoD* gene structure and sequencing results that confirmed biallelic frame-shifts of *MyoD* ORFs in one isolated *MyoD*^KO^ clone similar to our previous report [49]. Arrow points to a 1bp insertion; arrowhead points to a 1bp deletion. (**B**) Immunostaining results of MyoD (top) and myosin (bottom) of human WT and *MyoD*^KO^ myoblasts. MyoD staining confirmed the depletion of MyoD proteins in *MyoD*^KO^ cells. Scale bar, 100 μm. (**C**) Western blotting results that showed the absence of MyoG and myosin expression in human *MyoD*^KO^ myoblasts at 48 hours post differentiation. (**D**, **E**) qPCR results of human WT and *MyoD*^KO^ myoblasts with retroviral expression of *MyoD* or *MyoG*. Cells were differentiated for 48 hours. *n* = 3. Data are means ± SEM. *P < 0.05, **P < 0.01, ***P < 0.001. ns, not significant.
(TIF)

S3 Fig MyoG deletions from human myoblasts do not affect *DLL1* expression. (**A**) Human *MyoG* gene structure and an example of sequencing results similar to our previous report [49]. (**B**) Immunostaining result of MyoG and myosin to show the complete depletion of MyoG proteins and relatively mild defect of differentiation for one clonally derived human *MyoG*^KO^ myoblasts. Scale bar, 100 μm. (**C**) Quantifications of myoblast fusion. (**D**) qPCR result to show that *DLL1* expression was not significantly affected upon deletion of *MyoG* from human myoblasts. Data are means ± SEM. **P < 0.01, ***P < 0.001. ns, not significant.
(TIF)

S4 Fig Deletion of MyoD or MyoG affects Dll1 expression in mouse primary myoblasts. (**A**) Schematic of experiment design. (**B**) Immunostaining results of mouse primary myoblasts to show the expression levels of MyoD/MyoG before and after CRISPR treatments. Arrows point to cells that show cytoplasmic staining signals which are likely non-specific signals. (**C**) qPCR results of Dll1 in CRISPR treated mouse primary myoblasts. n = 3. Data are means ± SEM. ***P < 0.001.
(TIF)

S5 Fig Characterizations of *Dll1*^Δ232 /Δ232^ mouse. (**A**) Representative gel electrophoresis result of genotyping PCR of WT and *Dll1*^Δ232/Δ232^ mice. (**B**) Summary of *Dll1*^Δ232/Δ232^ genotyping results. (**C**) Schematic of experiment design and the timeline of treatments and tissue collections. (**D**) H&E staining results of cross-sections from control and injured tibialis anterior muscles. Arrows point to small regenerating myocytes. Scale bar, 200 μm.
(TIF)
