# Supplementary figures and images for: Feedback regulation of Notch signaling and myogenesis connected by MyoD–Dll1 axis
Source: PLoS Genet. 2021 Aug 9;17(8):e1009729. doi: 10.1371/journal.pgen.1009729 (PMC8376015; doi:10.1371/journal.pgen.1009729)

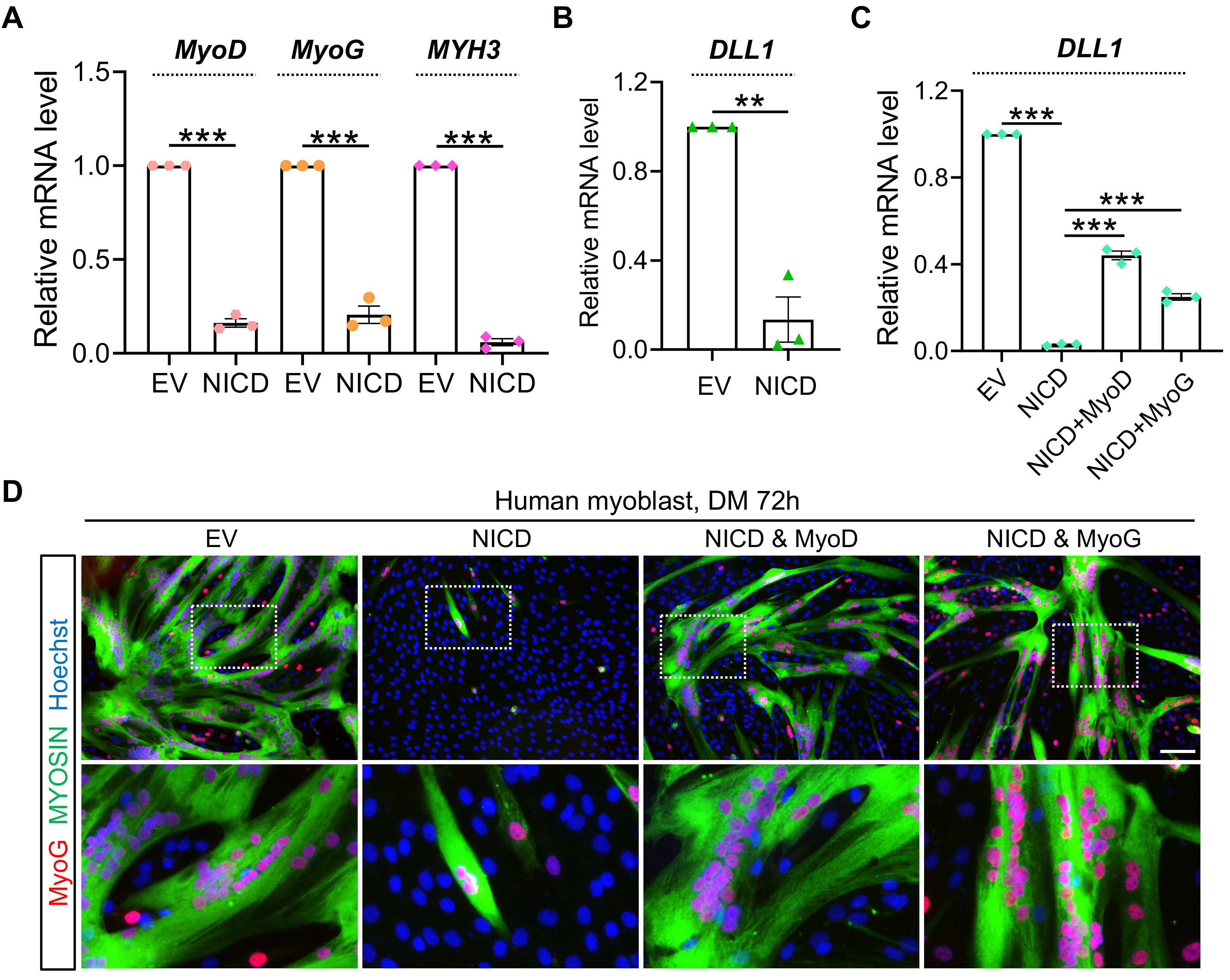

Supplement: S1 Fig — (A–C) qPCR results of myogenic markers (A), DLL1 (B, C) in human myoblasts with retroviral expression of NICD. Note that inhibitory effect of NICD on DLL1 expression was rescued by MyoD or MyoG expression (C). Cells were differentiated for 72 hours. n = 3. Data are means ± SEM. **P < 0.01, ***P < 0.001. (D) MyoG and myosin immunostaining results of human myoblasts after differentiation for 72 hours. Note that myogenic differentiation and fusion defects of NICD can be rescued by co-expression of MyoD or MyoG. Scale bar, 100 μm. (TIF) [file pgen.1009729.s001.tif]

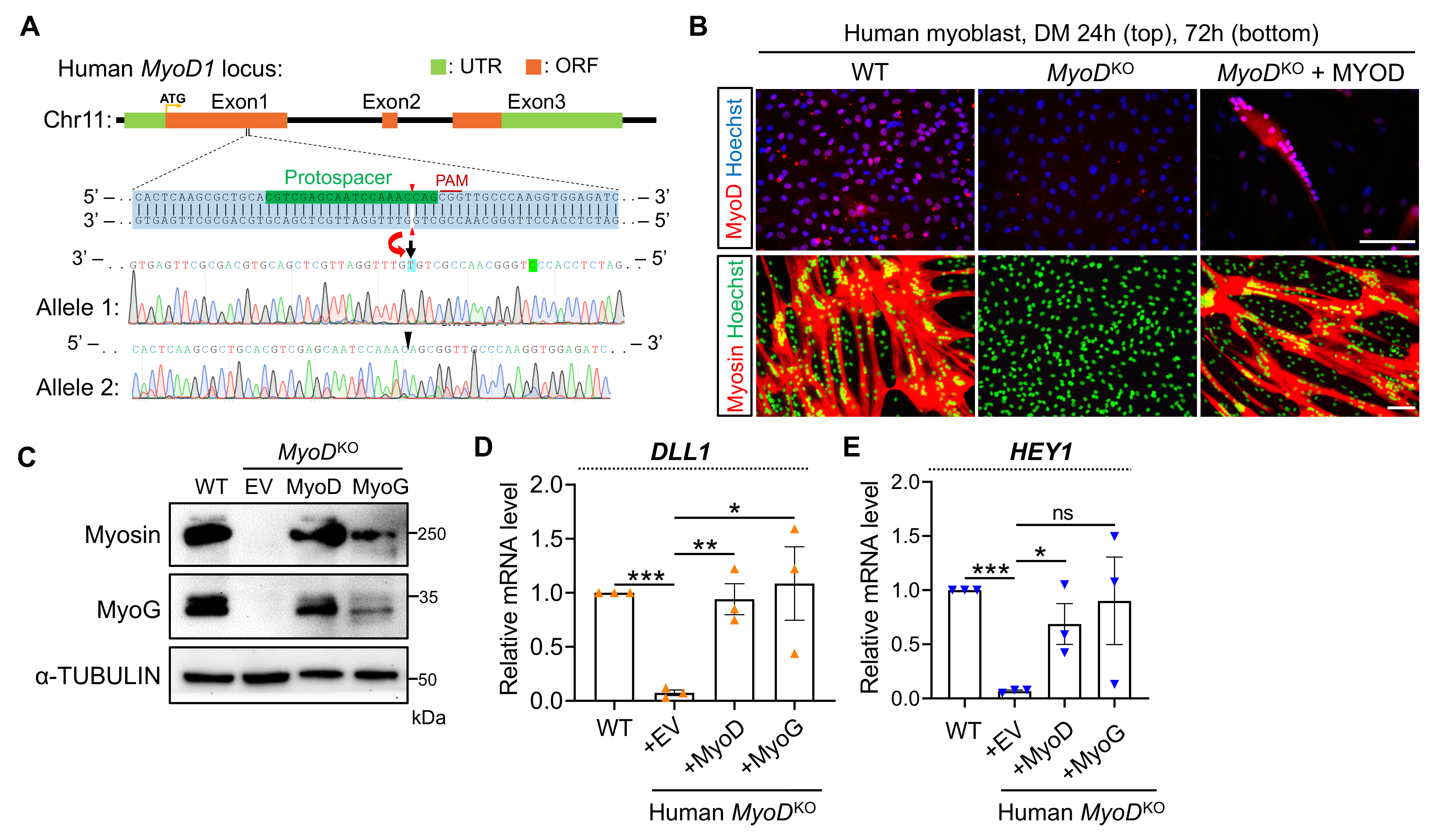

Supplement: S2 Fig — (A) Human MyoD gene structure and sequencing results that confirmed biallelic frame-shifts of MyoD ORFs in one isolated MyoDKO clone similar to our previous report [49]. Arrow points to a 1bp insertion; arrowhead points to a 1bp deletion. (B) Immunostaining results of MyoD (top) and myosin (bottom) of human WT and MyoDKO myoblasts. MyoD staining confirmed the depletion of MyoD proteins in MyoDKO cells. Scale bar, 100 μm. (C) Western blotting results that showed the absence of MyoG and myosin expression in human MyoDKO myoblasts at 48 hours post differentiation. (D, E) qPCR results of human WT and MyoDKO myoblasts with retroviral expression of MyoD or MyoG. Cells were differentiated for 48 hours. n = 3. Data are means ± SEM. *P < 0.05, **P < 0.01, ***P < 0.001. ns, not significant. (TIF) [file pgen.1009729.s002.tif]

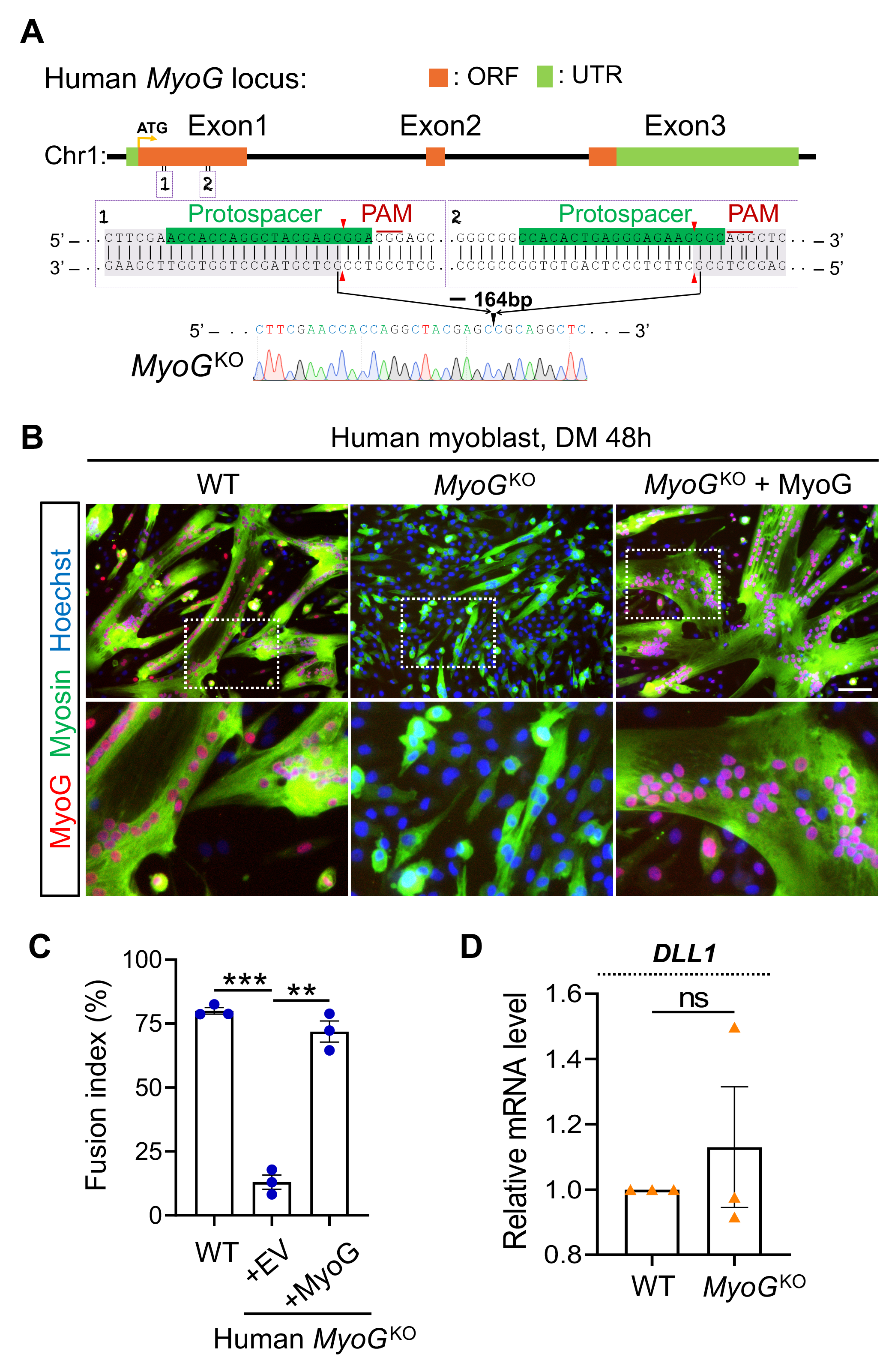

Supplement: S3 Fig — (A) Human MyoG gene structure and an example of sequencing results similar to our previous report [49]. (B) Immunostaining result of MyoG and myosin to show the complete depletion of MyoG proteins and relatively mild defect of differentiation for one clonally derived human MyoGKO myoblasts. Scale bar, 100 μm. (C) Quantifications of myoblast fusion. (D) qPCR result to show that DLL1 expression was not significantly affected upon deletion of MyoG from human myoblasts. Data are means ± SEM. **P < 0.01, ***P < 0.001. ns, not significant. (TIF) [file pgen.1009729.s003.tif]

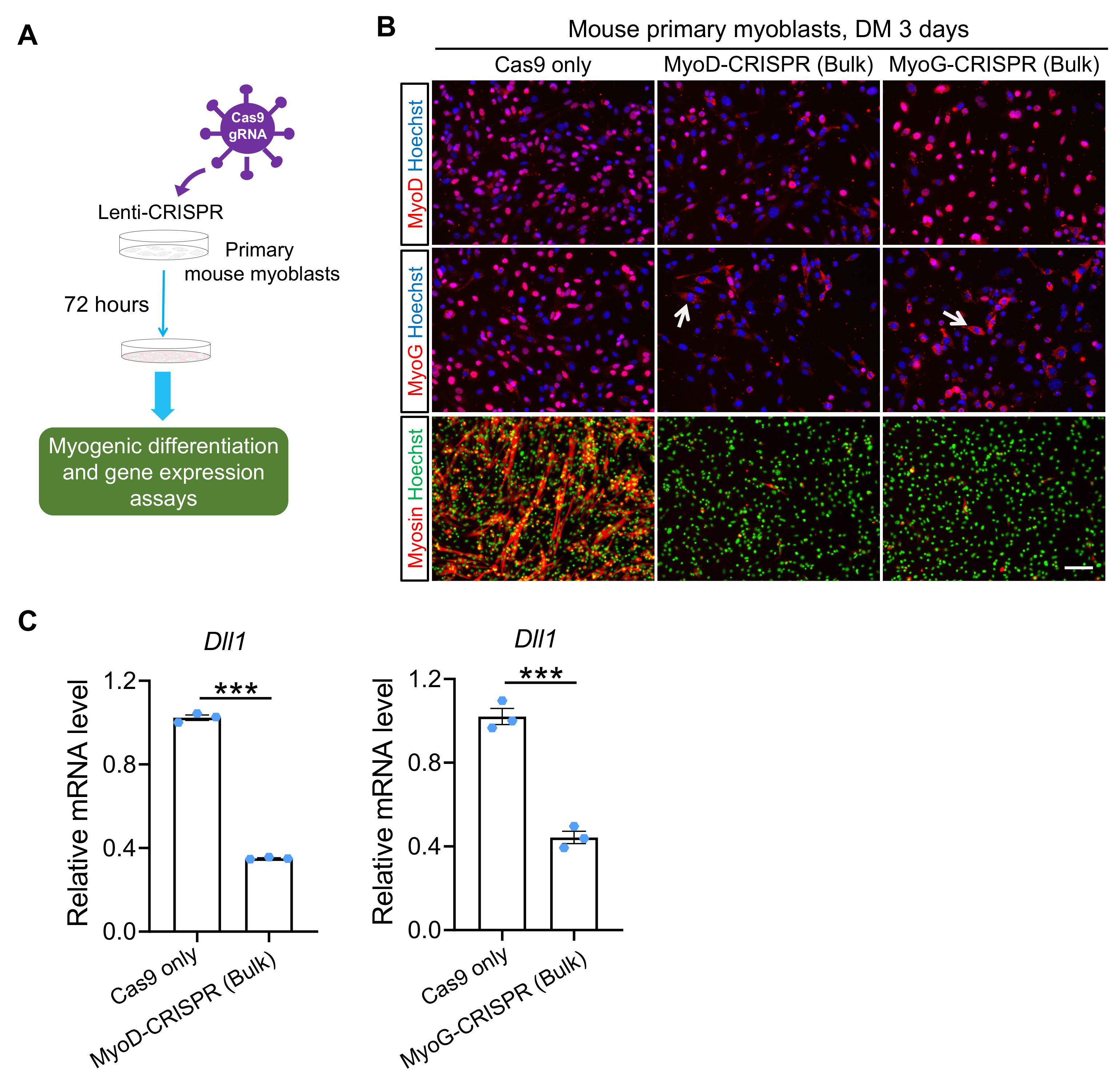

Supplement: S4 Fig — (A) Schematic of experiment design. (B) Immunostaining results of mouse primary myoblasts to show the expression levels of MyoD/MyoG before and after CRISPR treatments. Arrows point to cells that show cytoplasmic staining signals which are likely non-specific signals. (C) qPCR results of Dll1 in CRISPR treated mouse primary myoblasts. n = 3. Data are means ± SEM. ***P < 0.001. (TIF) [file pgen.1009729.s004.tif]

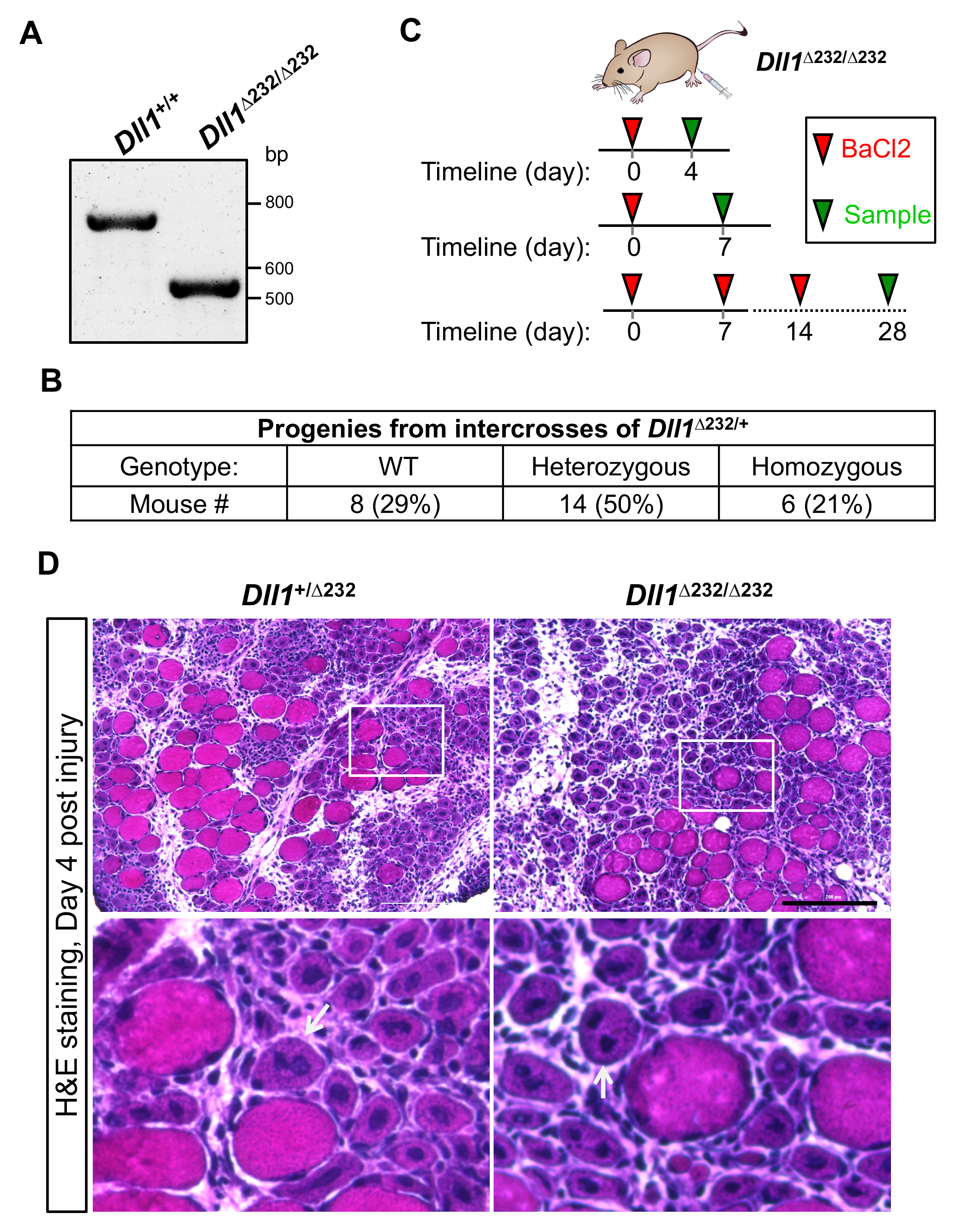

Supplement: S5 Fig — (A) Representative gel electrophoresis result of genotyping PCR of WT and Dll1Δ232/Δ232 mice. (B) Summary of Dll1Δ232/Δ232 genotyping results. (C) Schematic of experiment design and the timeline of treatments and tissue collections. (D) H&E staining results of cross-sections from control and injured tibialis anterior muscles. Arrows point to small regenerating myocytes. Scale bar, 200 μm. (TIF) [file pgen.1009729.s005.tif]
